# Demographic and Geographic Disparities in Atrial Fibrillation and Cirrhosis Mortality in the United States: A Twenty-Five-Year Analysis From 1999 to 2023

**DOI:** 10.14740/cr2194

**Published:** 2026-04-15

**Authors:** Ahmad Jalil, Fatima Rajab, Varshitha Bandi, Bilal Abaid, Karan Bhatt, Adharsh Ravindran, Vijay Chennareddy, Aleena Mujahid, Mahnoor Mehmood, Atif Ibrahim

**Affiliations:** a Department of Internal Medicine, Baptist Memorial Hospital–Oxford, Oxford, MS 38655, USA; b Department of Internal Medicine, King Edward Medical University, Lahore, Pakistan; c Department of Internal Medicine, University of Louisville, Louisville, KY, USA; d Department of Internal Medicine, Bay Area Hospital, Coos Bay, OR, USA; e Department of Internal Medicine, Texas Tech University Health Sciences Center, El Paso, TX, USA; f Department of Internal Medicine, Punjab Medical College, Faisalabad, Pakistan; g Department of Cardiology, North Mississippi Medical Center, Tupelo, MS, USA

**Keywords:** Cirrhosis, Atrial fibrillation, Mortality trends, CDC WONDER, Health disparities

## Abstract

**Background:**

Cirrhosis and atrial fibrillation (AF) are major public health conditions in the United States, each independently associated with substantial morbidity and mortality. Although AF is increasingly recognized in patients with cirrhosis, national mortality trends related to the coexistence of these conditions have not been well characterized.

**Methods:**

We conducted a nationwide ecological time-trend analysis using mortality data from the Centers for Disease Control and Prevention Wide-Ranging Online Data for Epidemiologic Research (CDC WONDER) from 1999 to 2023. Cirrhosis-related conditions and atrial fibrillation or flutter were identified using the International Classification of Diseases, 10th Revision (ICD-10) codes. Age-adjusted mortality rates (AAMRs) per 100,000 population were used and temporal trends were assessed using Joinpoint regression to estimate annual percent change (APC). Analyses were stratified by age, sex, race and ethnicity, geographic region, urban–rural classification, state, and place of death.

**Results:**

From 1999 to 2023, the AAMR associated with cirrhosis and AF increased from 0.2 to 1.7, representing more than an eightfold increase, with 39,957 total deaths recorded. Mortality rates were consistently higher in males than females, with a greater long-term increase among males (average annual percent change (AAPC) 9.25%; 95% confidence interval (CI), 8.35–10.25) compared with females (AAPC 8.61%; 95% CI, 6.18–11.14). Adults aged 65 years or older had substantially higher AAMRs than those aged 25–64 years, with significant increases observed after 2010 in both age groups. Hispanic or Latino individuals experienced the largest long-term increase in mortality (AAPC 9.33%; 95% CI, 7.81–10.89), followed by White individuals and Black or African American individuals. Regionally, the steepest increases occurred in the West (AAPC 10.22%; 95% CI, 9.04–11.33), while the Northeast showed the lowest growth. Urban–rural analyses through 2020 demonstrated the most rapid increases in noncore rural areas (AAPC 9.93%; 95% CI, 8.71–11.18).

**Conclusions:**

Mortality associated with the coexistence of cirrhosis and AF has increased substantially in the United States over the past 25 years, with accelerating trends and persistent demographic, geographic, and urban–rural disparities. These findings highlight an increasing public health burden requiring targeted surveillance and population level strategies.

## Introduction

Cirrhosis and atrial fibrillation (AF) represent two major public health challenges in the United States. Cirrhosis-related mortality has consistently been increasing in the United States since 2009, and it has increased by 65% from 1999 to 2016 [1]. At the same time, AF prevalence in the United States is projected to more than double, increasing from approximately 5.2 million cases in 2010 to 12.1 million by 2030 [2]. Apart from their independent morbidity and mortality, the intersection of these two conditions is increasingly recognized as clinically significant.

Cirrhosis is associated with increased cardiovascular mortality, particularly in patients with compensated disease, accounting for about 27% of deaths in patients with compensated nonalcoholic fatty liver disease (NAFLD)-related cirrhosis according to a study based in Ontario, Canada [3]. Similarly, mortality related to cardiovascular disease and cirrhosis is rising with a total of 374,090 deaths occurring between 1999 and 2019 in the United States [4]. Among cardiovascular diseases, atrial fibrillation and flutter are significantly recognized. According to a meta-analysis, the prevalence of AF in patients with cirrhosis was 5.0%, which increased to 7.4% after excluding transplant-only cohorts. Additionally, a statistically significant association has been reported between AF and increased mortality risk in patients with cirrhosis [5]. Atrial fibrillation and flutter are increasingly recognized in patients with cirrhosis and there could be several reasons behind this. In patients with cirrhosis, AF can arise from a combination of cirrhotic cardiomyopathy with high cardiac output and elevated atrial pressures causing left atrial dilation and wall stress, together with electrolyte imbalances, hepatorenal syndrome–related hemodynamic derangements, increased serum bile acids, metabolic abnormalities, systemic inflammatory activation, and atrial interstitial fibrosis, all of which promote an arrhythmogenic substrate [6, 7]. Inflammation has been found to be associated with the generation and perpetuation of AF, and several inflammatory markers are elevated in patients with cirrhosis [8].

Despite the clinical significance of AF in cirrhotic patients and the well-documented mortality burden of each condition independently, national trends in mortality specifically related to the co-occurrence of AF and cirrhosis have not been examined. This study aims to characterize mortality trends related to AF and cirrhosis in the United States from 1999 to 2023 using the Centers for Disease Control and Prevention Wide-Ranging Online Data for Epidemiologic Research (CDC WONDER) mortality data. Specifically, we will examine temporal trends in age-adjusted mortality rates (AAMRs), identify demographic disparities by age, sex, and race/ethnicity, assess geographic variations across states and regions. By identifying these patterns, this analysis will provide important insights into the epidemiology of this high-risk population and help in developing evidence-based strategies to reduce preventable deaths.

## Materials and Methods

### Study setting and population

In this nationwide ecological time-trend analysis, an in-depth search was conducted on combined cirrhosis- and AF-related mortality in the US population from 1999 to 2023, and death certificate–based data were retrieved from the CDC WONDER underlying cause of death files for age ≥ 25 year.

The study used diagnostic codes from the International Classification of Diseases, 10th Revision (ICD-10) to identify cirrhosis-related conditions, including I85.0 (esophageal varices with bleeding), I85.9 (esophageal varices without bleeding), I86.4 (gastric varices), K70.2 (alcoholic fibrosis and sclerosis of liver), K70.3 (alcoholic cirrhosis of liver), K71.7 (toxic liver disease with fibrosis and cirrhosis of liver), K74.0 (hepatic fibrosis), K74.1 (hepatic sclerosis), K74.2 (hepatic fibrosis with hepatic sclerosis), K74.3 (primary biliary cirrhosis), K74.4 (secondary biliary cirrhosis), K74.5 (biliary cirrhosis, unspecified), K74.6 (other and unspecified cirrhosis of liver), K76.6 (portal hypertension), and K76.7 (hepatorenal syndrome).

Atrial fibrillation and flutter were identified using ICD-10 code I48. Multiple causes of death files were used to extract deaths related to cirrhosis and AF among adults aged ≥ 25 years, using ICD-10 codes. CDC dataset has been used in prior studies for mortality analyses of cirrhosis and cardiovascular conditions. Information from death certificates from all 50 states and the District of Columbia is included in this collection [9].

### Data abstraction

The dataset included year of death, population size, demographic characteristics, urban–rural classification, geographic segmentation, and location of death, including hospitals, nursing homes, long-term care facilities, households, and hospices. Joinpoint Trend Analysis Software was used to assess changes in temporal trends by estimating annual percent change (APC) for individual trend segments and average annual percent change (AAPC) across the overall study period.

Analyses were stratified by variables available in CDC WONDER, including age, sex, geography, urban–rural classification, and race/ethnicity. Age was further stratified into 25–64 years and ≥ 65 years. Sex was categorized as male or female. Race and ethnicity were classified as non-Hispanic White (NHW), non-Hispanic Black (NHB) or African American, non-Hispanic American Indian or Alaska Native (NHAI), non-Hispanic Asian or Pacific Islander (NHA), and Hispanic or Latino.

Location of death, including medical facilities, home, hospice, and nursing home/long-term care facilities, was also evaluated. This information was obtained from CDC WONDER [9].

Urban–rural status was assessed using the 2013 US Census classification in accordance with the National Center for Health Statistics Urban–Rural Classification Scheme, applied uniformly across all study years (1999–2023) to ensure consistency and comparability. Geographic regions were categorized as West, Northeast, South, and Midwest, according to the United States Census Bureau [10, 11].

Mortality rates were analyzed using two age groups (25–64 years and ≥ 65 years) to reflect the substantially higher burden of cirrhosis and AF–related mortality in older adults. Analyses involving states and urbanization were restricted to 1999–2020, corresponding to the most recent years for which these variables were available in CDC WONDER in consolidated form. Certain race/ethnicity subgroups, including NHAI and NHA, were not analyzed because small cell counts precluded reliable estimates.

### Statistical analysis

We calculated crude death rates and AAMRs per 100,000 population from 1999 to 2023 using CDC WONDER to examine temporal trends. Rates were further stratified by sex, year, age group, race/ethnicity, and state, with 95% confidence intervals (CIs) reported.

AAMRs were calculated by standardizing cirrhosis- and AF-related deaths to the 2000 US standard population [12]. Crude mortality rates were calculated by dividing the number of cirrhosis- and AF-related deaths by the corresponding US population for each year [9].

APC with 95% CIs was estimated using the Joinpoint Regression Program (Joinpoint version 5.3.0.0; National Cancer Institute) with permutation testing, allowing a maximum of three joinpoints [13]. Using two-tailed testing, APC estimates were considered statistically significant when the slope differed from zero, with a P value < 0.05.

### Institutional Review Board (IRB) approval

This study used deidentified, government-issued public-use data obtained from the CDC WONDER database and was therefore exempt from IRB review, as it did not involve human subjects as defined by federal regulations.

### Ethical compliance with human/animal study

This study was conducted in compliance with the ethical standards of the responsible institution and with the principles outlined in the Declaration of Helsinki. The analysis utilized publicly available, deidentified data and did not involve direct interaction with human participants or animals. The study follows the Strengthening the Reporting of Observational Studies in Epidemiology (STROBE) guidelines for reporting.

## Results

**Figure 1:**
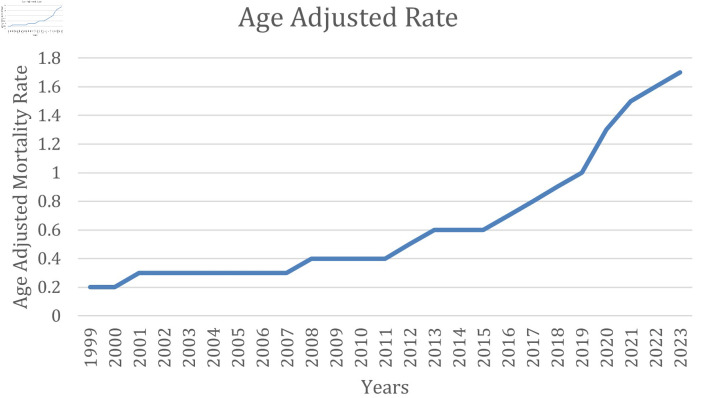
AAMR due to AF and cirrhosis and AF over 25 years. AAMR: age-adjusted mortality rate; AF: atrial fibrillation.

The AAMR due to cirrhosis and AF increased from 0.2 (95% CI, 0.2–0.3) in 1999 to 1.7 (95% CI, 1.7–1.8) in 2023 (Fig. 1), with a total of 39,957 deaths and average age-adjusted mortality of 0.636 recorded between 1999 and 2023.

**Figure 2:**
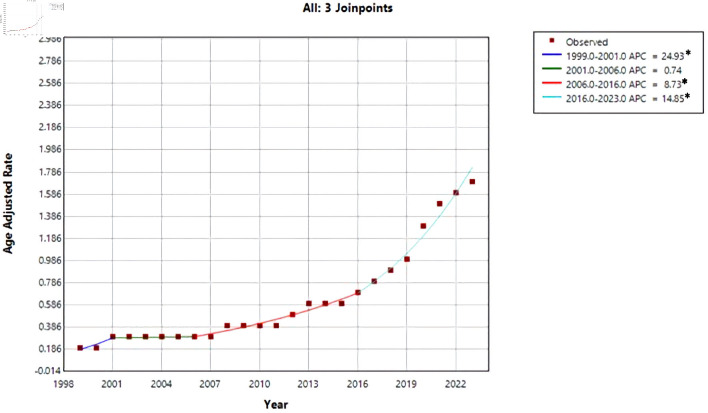
Trend of AAMR due to AF and cirrhosis over the years. *Indicates that annual percent change (APC) is significantly different from zero at the α = 0.05 level. Final selected model: three joinpoints. AAMR: age-adjusted mortality rate; AF: atrial fibrillation.

The Joinpoint analysis for cirrhosis and AF–related mortality demonstrates an overall increasing trend with periods of acceleration, particularly after 2016 (Fig. 2). From 1999 to 2001, mortality increased sharply, with a significant APC of 24.93%, indicating an early and rapid rise in mortality during this period. This was followed by a plateau phase from 2001 to 2006, during which the trend stabilized and was not statistically significant (APC = 0.74%).

Beginning in 2006, mortality rates resumed an upward trajectory, with a significant increase from 2006 to 2016 (APC = 8.73%), reflecting a sustained rise over the following decade. The most pronounced escalation occurred in the most recent period, from 2016 to 2023, during which mortality increased at a significantly higher rate (APC = 14.85%), indicating a marked acceleration in cirrhosis and AF–related deaths in recent years. Overall, these findings highlight a worsening long-term mortality trend, with particularly rapid increases observed in the last decade.

### Gender stratification

**Figure 3:**
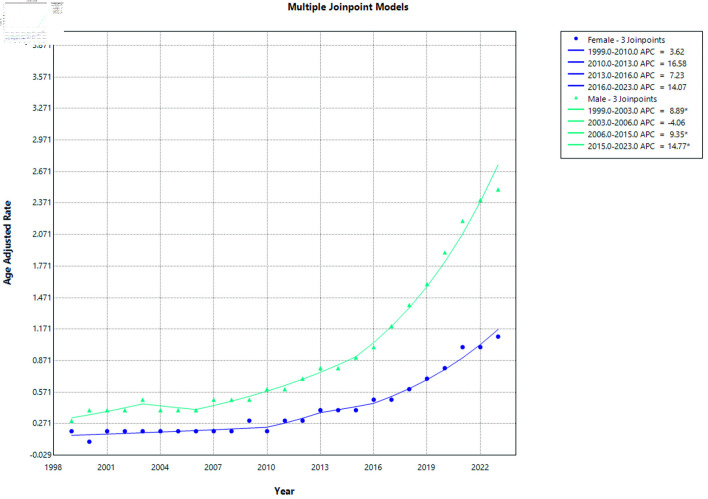
AF and cirrhosis–related mortality stratified by gender. APC: annual percent change; AF: atrial fibrillation.

From 1999 to 2023, males consistently demonstrated higher AAMRs compared with females, as shown in Figure 3.

Among females, the AAMR showed a gradual upward trend over the study period. From 1999 to 2010, the mortality rate increased modestly (APC = 3.62%; 95% CI −17.71 to 38.89; P = 0.5419). This was followed by a non-significant increase from 2010 to 2013 (APC = 16.58%; 95% CI, −13.23 to 30.00; P = 0.5027) and from 2013 to 2016 (APC = 7.23%; 95% CI, −8.48 to 33.39; P = 0.4499). From 2016 to 2023, the trend continued to increase but did not reach statistical significance (APC = 14.07%; 95% CI, −14.29 to 44.43; P = 0.144) (Fig. 1 and Supplementary Material 1, cr.elmerpub.com). However, when analyzed collectively, the increase from 2010 to 2023 was statistically significant (APC = 12.56%).

In males, mortality rates demonstrated multiple statistically significant periods of increase. A significant rise in age-adjusted mortality was observed from 1999 to 2003 (APC = 8.89%; 95% CI, 2.32 to 21.89; P = 0.012), followed by a non-significant decline from 2003 to 2006 (APC = −4.06%; 95% CI, −8.31 to 13.11; P = 0.3959). Subsequently, mortality increased significantly from 2006 to 2015 (APC = 9.35%; 95% CI, 4.26 to 18.14; P = 0.008) and continued to rise significantly from 2015 to 2023 (APC = 14.77%; 95% CI, 8.00 to 24.46; P = 0.018) (Fig. 3 and Supplementary Material 2, cr.elmerpub.com).

From 1999 to 2023, AAMRs increased significantly in both sexes (all P < 0.0001) (Supplementary Materials 2, 3, cr.elmerpub.com). Males experienced a slightly greater long-term increase in mortality (AAPC = 9.25%; 95% CI, 8.35–10.25) compared with females (AAPC = 8.61%; 95% CI, 6.18–11.14). Overall, these findings indicate a consistent upward trend in mortality for both sexes, with a modestly steeper rise observed among males.

### Racial stratification

**Figure 4:**
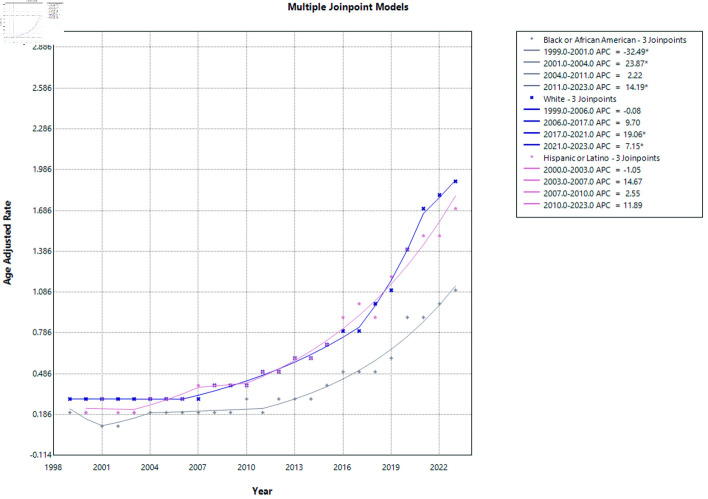
AF and cirrhosis–related mortality stratified by race. APC: annual percent change; AF: atrial fibrillation.

From 1999 to 2023, AAMRs increased significantly across all racial and ethnic groups examined (all P < 0.0001) (Fig. 4 and Supplementary Materials 4–6, cr.elmerpub.com). Despite the absence of statistically significant changes within individual joinpoint segments, Hispanic or Latino individuals demonstrated a significant overall increase in mortality over the study period, as reflected by the AAPC. White individuals also demonstrated a substantial and statistically significant rise in mortality (AAPC = 8.01%; 95% CI, 7.54–8.57). In contrast, Black or African American individuals exhibited a comparatively lower, though still significant, increase in mortality over the study period (AAPC = 6.90%; 95% CI, 5.45–8.72). Overall, these findings indicate a consistent upward trend in mortality across racial and ethnic groups, with the most rapid long-term growth observed among Hispanic or Latino populations.

For NHB or African American individuals, mortality rates showed marked variability over the study period. From 1999 to 2001, there was a significant decline in mortality (APC = −32.49; 95% CI, −44.36 to −11.91; P = 0.0156). This was followed by a significant increase from 2001 to 2004 (APC = 23.87; 95% CI, 6.25 to 39.94; P = 0.0060). From 2004 to 2011, mortality rates remained relatively stable with no statistically significant change (APC = 2.22; 95% CI, −12.36 to 23.57; P = 0.9158). However, from 2011 to 2023, a significant upward trend was observed (APC = 14.19; 95% CI, 3.89 to 21.34; P = 0.038), indicating a sustained increase in mortality in recent years for this population.

Among NHW individuals, mortality rates were initially stable from 1999 to 2006 (APC = −0.08; 95% CI, −2.47 to 2.11; P = 0.9054). This was followed by a borderline increase from 2006 to 2017 that did not reach statistical significance (APC = 9.70; 95% CI, −1.19 to 12.45; P = 0.0588). A pronounced and statistically significant increase was observed from 2017 to 2021 (APC = 19.06; 95% CI, 7.20 to 24.30; P < 0.0001), which persisted from 2021 to 2023 (APC = 7.15; 95% CI, 1.20 to 16.32; P = 0.0200), confirming a recent acceleration in mortality rates among White individuals.

When stratified by ethnicity, Hispanic or Latino individuals demonstrated no statistically significant changes across the study period. From 2000 to 2003, mortality rates showed a non-significant decline (APC = −1.05; 95% CI, −17.53 to 12.77; P = 0.8618), followed by non-significant increases from 2003 to 2007 (APC = 14.67; 95% CI, −3.11 to 29.99; P = 0.1144) and from 2007 to 2010 (APC = 2.55; 95% CI, −4.97 to 26.32; P = 0.3787). From 2010 to 2023, mortality rates continued to increase, although this trend did not reach statistical significance (APC = 11.89; 95% CI, −6.19 to 29.66; P = 0.0856). The lack of significance in segment-specific APCs likely reflects wide CIs and shorter interval lengths.

### Age-based stratification

**Figure 5:**
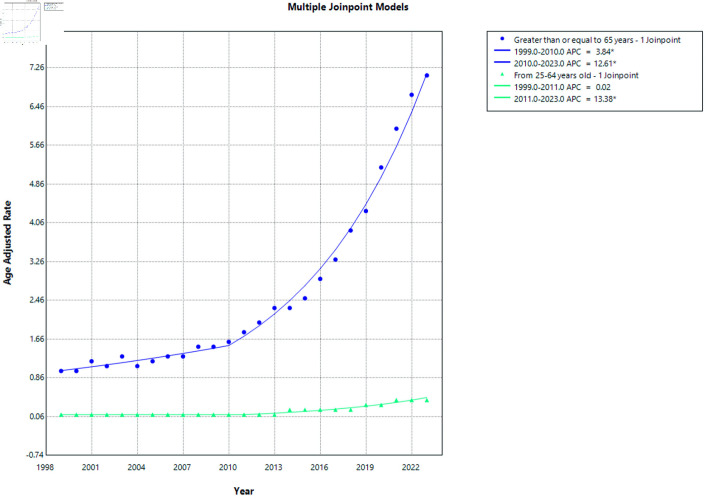
AF and cirrhosis–related mortality stratified by age. APC: annual percent change; AF: atrial fibrillation.

From 1999 to 2023, individuals aged ≥ 65 years consistently demonstrated substantially higher AAMRs compared with those aged 25–64 years, as shown in Figure 5 with their ACP shown here (Supplementary Materials 7, 8, cr.elmerpub.com).

Among individuals aged ≥ 65 years, the AAMR showed a steady increase over the study period. A significant rise was observed from 1999 to 2010 (APC = 3.84%; P < 0.028), followed by a more pronounced and statistically significant increase from 2010 to 2023 (APC = 12.61%; P <0.0001), as illustrated in Figure 5. This pattern indicates an acceleration in mortality rates in the older population during the latter part of the study period.

In contrast, individuals aged 25–64 years exhibited relatively stable mortality rates initially. From 1999 to 2011, the trend remained essentially unchanged (APC = 0.02%; P > 0.989). However, a statistically significant increase in AAMRs was observed from 2011 to 2023 (APC = 13.38%; P < 0.0001), as shown in Figure 5, indicating a recent upward shift in mortality among the younger age group.

### Mortality trends by census region

**Figure 6:**
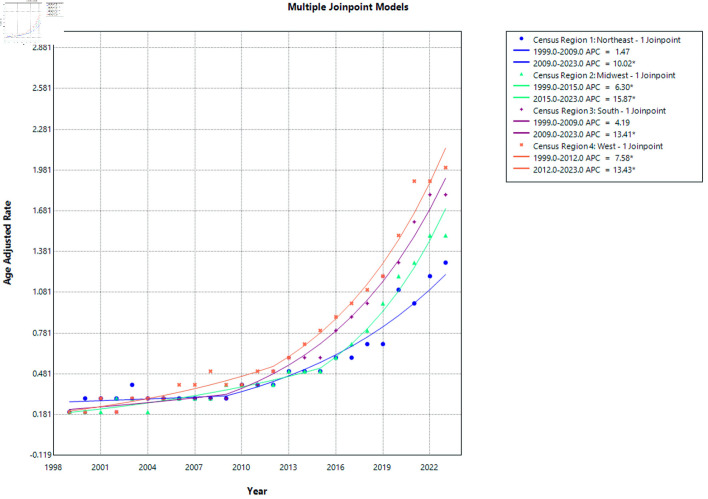
AF and cirrhosis–related mortality stratified by census region. APC: annual percent change; AF: atrial fibrillation.

From 1999 to 2023, AAMRs from cirrhosis and AF increased significantly across all US Census regions (all P < 0.0001) with their APC and AAPC mentioned here (Supplementary Materials 9–11, cr.elmerpub.com). The West experienced the steepest long-term increase in mortality (AAPC = 10.22%; 95% CI, 9.04–11.33), followed by the South (AAPC = 9.47%; 95% CI, 8.51–10.45) and the Midwest (AAPC = 9.40%; 95% CI, 8.35–10.38). In contrast, the Northeast demonstrated a comparatively lower, though still significant, rise in mortality (AAPC = 6.37%; 95% CI, 5.23–7.48). Overall, these findings indicate a consistent upward trend nationwide, with more rapid long-term growth observed outside the Northeast. The mortality trends are shown in Figure 6.

In the Northeast, mortality rates remained relatively stable from 1999 to 2009, with no statistically significant change (APC = 1.47; 95% CI, −5.09 to 4.34; P = 0.476). However, from 2009 to 2023, a significant and sustained increase in mortality was observed (APC = 10.02; 95% CI, 8.14 to 13.70; P < 0.0001), indicating a marked upward trend in the later years.

In the Midwest, mortality rates increased significantly throughout the study period. From 1999 to 2015, there was a statistically significant rise in mortality (APC = 6.30; 95% CI, 4.08 to 7.67; P = 0.0036), followed by an even steeper and highly significant increase from 2015 to 2023 (APC = 15.87; 95% CI, 11.85 to 24.98; P < 0.0001), representing the most pronounced regional acceleration in mortality.

In the South, mortality rates showed a non-significant upward trend from 1999 to 2009 (APC = 4.19; 95% CI, −1.06 to 6.82; P = 0.0788). This was followed by a significant and sustained increase from 2009 to 2023 (APC = 13.41; 95% CI, 11.66 to 16.23; P < 0.0001), highlighting a substantial rise in mortality over the past decade.

In the West, mortality rates increased significantly from 1999 to 2012 (APC = 7.58; 95% CI, 0.33 to 9.46; P = 0.0468). This upward trend persisted and intensified from 2012 to 2023, with a statistically significant increase in mortality (APC = 13.43; 95% CI, 10.94 to 23.63; P = 0.0032), confirming a continued escalation in recent years.

### Urbanization trends (2013)

**Figure 7:**
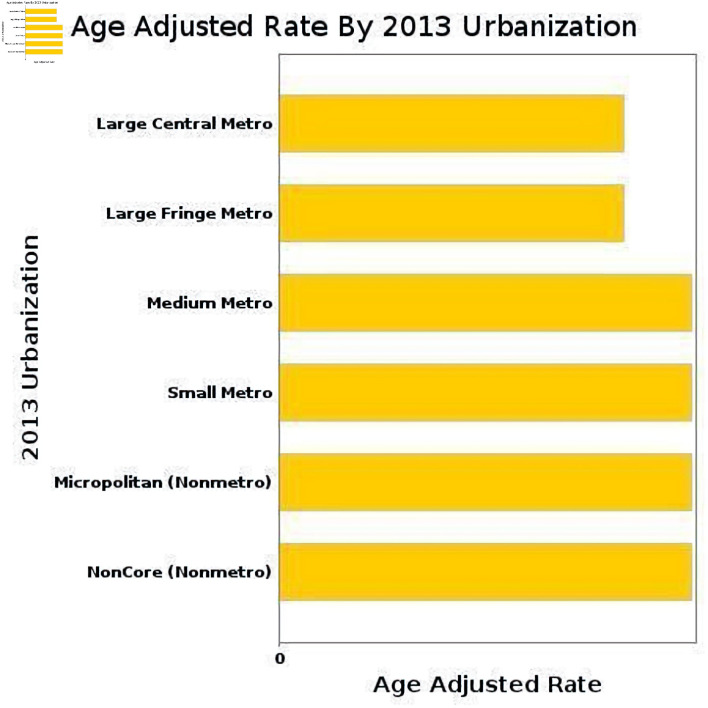
Overall AAMR of urban–rural classification due to AF and cirrhosis. AAMR: age-adjusted mortality rate; AF: atrial fibrillation.

According to 2013 Urban–Rural classification, the overall AAMR are depicted in Figure 7.

**Figure 8:**
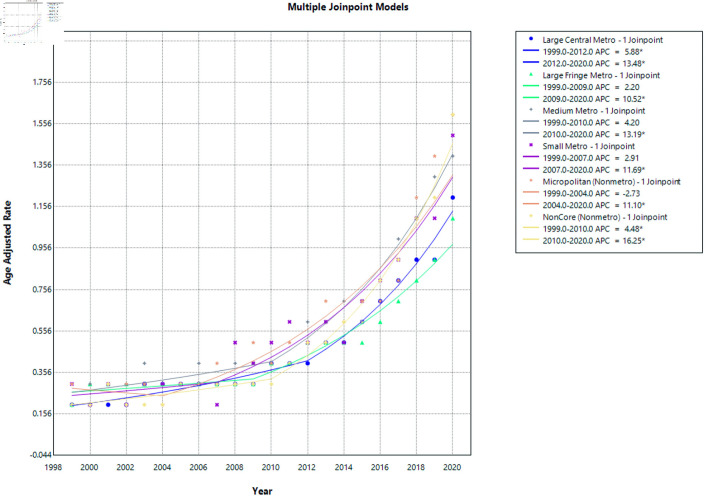
Urban–rural trends in AF and cirrhosis related mortality. APC: annual percent change; AF: atrial fibrillation.

The urban–rural trends in cirrhosis and AF–related mortality (Fig. 8) demonstrate increasing mortality across all urban and nonmetropolitan classifications in recent years, with particularly steep rises observed in noncore and metropolitan areas with their APC, and AAMR shown here (Supplementary Materials 12–14, cr.elmerpub.com).

In large central metropolitan areas, mortality increased significantly from 1999 to 2012 (APC = 5.88; 95% CI, 2.51 to 7.40; P = 0.020) and accelerated further from 2012 to 2020 (APC = 13.48; 95% CI, 10.27 to 21.96; P < 0.0001), indicating a sustained and intensifying upward trend.

In large fringe metropolitan areas, mortality rates remained relatively stable from 1999 to 2009 with no statistically significant change (APC = 2.20; 95% CI, −3.75 to 4.82; P = 0.287). However, a significant increase was observed from 2009 to 2020 (APC = 10.52; 95% CI, 8.21 to 16.25; P = 0.0008), reflecting a notable rise in mortality during the later period.

For medium metropolitan areas, mortality showed a non-significant upward trend from 1999 to 2010 (APC = 4.20; 95% CI, −0.48 to 6.54; P = 0.066), followed by a significant increase from 2010 to 2020 (APC = 13.19; 95% CI, 10.34 to 19.26; P < 0.0001), indicating a marked escalation in mortality rates over the past decade.

In small metropolitan areas, mortality rates were stable from 1999 to 2007 (APC = 2.91; 95% CI, −15.56 to 8.18; P = 0.611). This was followed by a statistically significant increase from 2007 to 2020 (APC = 11.69; 95% CI, 8.95 to 29.53; P = 0.015), demonstrating rising mortality in smaller urban centers.

Among micropolitan (nonmetropolitan) areas, mortality showed no significant change from 1999 to 2004 (APC = −2.73; 95% CI, −24.41 to 9.35; P = 0.677). However, a significant increase was observed from 2004 to 2020 (APC = 11.10; 95% CI, 7.93 to 27.27; P = 0.039), indicating growing mortality burden in micropolitan communities.

In noncore (rural) areas, mortality increased significantly throughout the study period. From 1999 to 2010, mortality rose (APC = 4.48; 95% CI, 0.73 to 6.77; P = 0.030), followed by a steeper and highly significant increase from 2010 to 2020 (APC = 16.25; 95% CI, 13.25 to 21.27; P < 0.0001), representing the fastest growth in mortality among all urban–rural categories.

From 1999 to 2020, AAMRs increased significantly across all urban–rural categories (all P < 0.0001). Noncore (rural) areas experienced the greatest rise (AAPC = 9.93%; 95% CI, 8.71 to 11.18), followed by large central metropolitan (AAPC = 8.71%; 95% CI, 7.69 to 9.70), medium metropolitan (AAPC = 8.39%; 95% CI, 7.14 to 9.63), and small metropolitan areas (AAPC = 8.26%; 95% CI, 6.28 to 10.47). Large fringe metropolitan areas showed the lowest, though still significant, increase (AAPC = 6.48%; 95% CI, 5.36 to 7.67). Overall, mortality rose consistently across the urban–rural continuum, with the steepest long-term growth in rural regions.

### Cirrhosis and AF–related mortality stratified by geographic region

**Figure 9:**
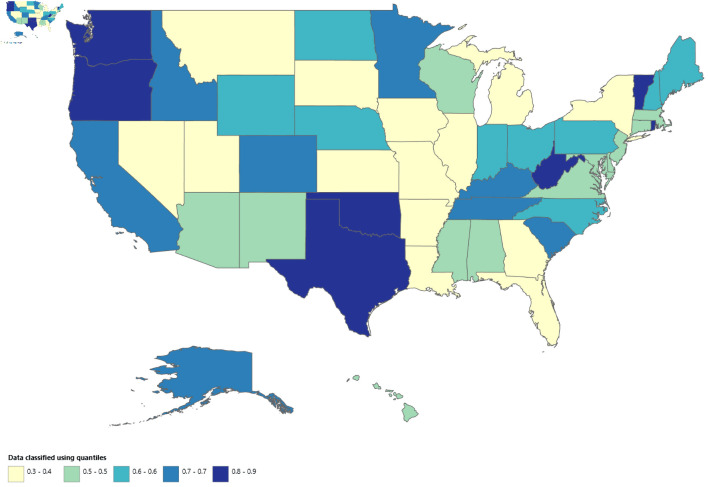
Map showing geographic distribution of AF and cirrhosis–related mortality from 1999 to 2020. AF: atrial fibrillation.

The state-wise trends in cirrhosis and AF–related mortality reveal marked variation across the United States (Fig. 9 and Supplementary Material 15, cr.elmerpub.com). Based on AAMRs from 1999 to 2020, Vermont and Oregon exhibited the highest average AAMR (at 0.9), which increased to 3.3 and 3.9, respectively, in 2023, indicating the greatest overall burden among US states during the study period. In addition, Texas, Oklahoma, and West Virginia consistently appear within the higher mortality categories on the map.

In contrast, Utah reported the lowest AAMR from 1999 to 2020, with an AAMR of 0.3 (95% CI, 0.2 to 0.4). Several other states, including New York, Louisiana, and Illinois, also demonstrated low mortality rates. Overall, these findings highlight substantial geographic heterogeneity in cirrhosis and AF–related mortality across states, with certain regions experiencing a disproportionately higher burden compared with others.

### Place of death

**Figure 10:**
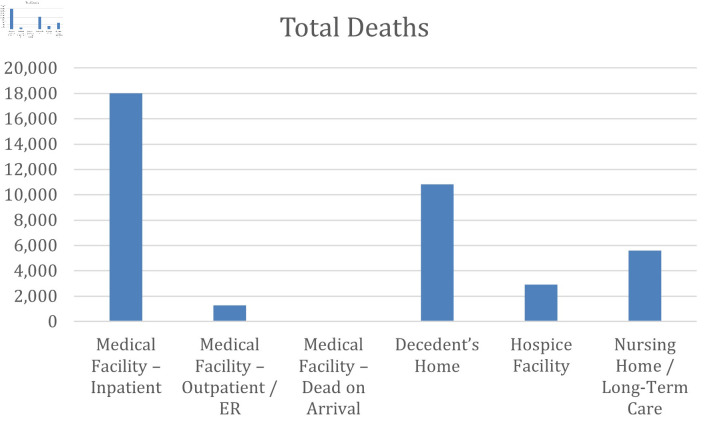
Distribution of total deaths due to AF and cirrhosis by place of death. The bar chart displays the total number of deaths on the y-axis stratified by place of death on the x-axis. AF: atrial fibrillation.

Analyzing the number of deaths due to cirrhosis and AF from 1999 to 2023 (Fig. 10 and Supplementary Material 16, cr.elmerpub.com), the largest proportion of deaths occurred in inpatient medical facilities, followed by deaths at the decedent’s home and in nursing homes or long-term care facilities. Fewer deaths were observed in hospice facilities, while deaths occurring in outpatient or emergency department settings and on arrival were relatively uncommon. These findings represent absolute distributions of deaths by place of occurrence and do not reflect AAMRs or relative risk across settings.

## Discussion

Our study demonstrates an overall upward trend in mortality rates associated with combined cirrhosis and AF from 1999 to 2023, with increases observed across multiple demographic groups, geographic regions, and urbanization categories in the United States. The AAMR increased more than eightfold, from 0.2 per 100,000 in 1999 to 1.7 per 100,000 in 2023, with the most pronounced escalation occurring from 2016 to 2023 (APC = 14.85%). Males consistently demonstrated higher mortality rates as compared to females throughout the study period. Among different racial and ethnic groups, Hispanic or Latino individuals experienced the largest long-term increase in mortality, while advanced age was strongly associated with elevated mortality risk. Geographically, the West showed the steepest increases, and noncore rural areas experienced the most rapid mortality growth among all urbanization categories.

With these trends, it is evident that AF is increasingly recognized in patients with cirrhosis. The prevalence of AF in patients with cirrhosis ranges between 6.6% and 14.2%, substantially higher than the general population [14]. Cirrhosis is an independent risk factor for AF development, with a hazard ratio of 1.46 (95% CI, 1.18 to 1.80) after multivariate adjustment, particularly in younger men without traditional cardiovascular comorbidities [15].

Portal hypertension in cirrhosis creates a hyperdynamic circulatory state characterized by increased cardiac output, splanchnic vasodilation, elevated atrial pressures and altered wall stress [7, 16]. Atrial stretch activates the renin-angiotensin-aldosterone system, which generates multiple downstream profibrotic factors, including transforming growth factor-beta 1 [17]. The renin-angiotensin-aldosterone system regulates blood pressure and, when activated, promotes vascular smooth muscle constriction, activates fibroblasts (increasing atrial interstitial collagen), and increases reactive oxygen species in the sympathetic nervous system [18].

Studies providing AF- and cirrhosis-related mortality data are limited. However, focusing on individual risk factors, we have found that AF is associated with a substantially increased risk of death, with studies showing roughly a 1.5–2-fold higher mortality compared with individuals without AF. Although women with AF experience higher relative mortality than men, with meta-analytic data indicating about a 12% higher all-cause mortality risk in women versus men, the overall population-level mortality burden remains greater in men, because AF is more common and incident in males across most age groups and regions [18–21]. Our analysis also shows the age-standardized mortality rate from AF and cirrhosis increased more in males as compared to females. Similar to our analysis, Aziz et al also noted higher AAMRs in males for combined heart failure and cirrhosis [22].

Lower cirrhosis mortality rates in females compared to males result from a combination of protective biological factors, particularly sex hormones, and lower exposure to high-risk behaviors, though these advantages are modified by age and etiology-specific factors. Estrogens protect women from visceral obesity, insulin resistance, non-alcoholic fatty liver disease, cirrhosis, and hepatocellular carcinoma [23].

Black and Hispanic patients with AF are often found to have higher mortality rates when compared to White population. This difference mainly stems from the higher risk of stroke development and/or coronary artery disease in the Black and Hispanic patients due to their relatively lower healthcare access and hence lesser percentage of the population being on anticoagulation [24]. In our study, the mortality of patients with AF and cirrhosis combined has shown a consistent increase across all racial groups, with the most recent and sharpest rise being in White and Hispanic individuals, as compared to Black patients who have a later but sustained increase around 2011. Our observation of a steeper long-term increase in AF-cirrhosis mortality among White than Black individuals is consistent with broader liver disease trends, where age-standardized liver mortality has decreased modestly in Black adults but increased in White adults, with the Black-to-White rate ratio falling from 1.26 to 0.79 over the past two decades. Prior work has attributed this shift in part to rising alcohol-related and metabolic dysfunction–associated steatotic liver disease deaths in White populations, which may similarly contribute to the sharper mortality increase we observed in White patients with concomitant AF and cirrhosis [25].

AF markedly increases the mortality rates by 1.8–2.6-fold in patients older than 60 years, which could be attributed to age-related cardiac remodeling and fragility [26]. In patients with liver cirrhosis, AF is associated with increased mortality in hospitalized patients and the 4-year all-cause mortality with a hazard ratio of 1.5 [27]. Across our study period, age-adjusted mortality was consistently higher in adults of age 65 or older, with a clear acceleration observed from 2010. In comparison to this, adults aged 25–64 years had stable mortality trends initially; however, since 2011, there has been an upward shift in AF-related mortality, which could be attributed to the growing burden of modifiable risk factors. The study by Aziz et al revealed similar age-related mortality, with higher rates in older age groups and sharp recent increases [22]. However, our study highlights synchronized post-2010/2011 surges across 25–64 year and ≥ 65-year age groups, which is unique to the AF–liver comorbidity.

AF shows marked regional disparities across the census divisions in the United States, with the West and Midwest regions having higher AAMRs. The South has demonstrated very steep upward trends, while the Northeast has an overall lower AAMRs, most likely reflecting the differences in cardiovascular risk factor prevalence, healthcare access, and urbanization [28]. In contrast, cirrhosis-related mortality is highest in the South, followed by the West, with the lowest being in the Northeast, which could be attributed to the alcohol-related diseases and rural burdens [29]. Our AF-cirrhosis mortality analysis showed a steep increase in the West and South, with all regions showing significant post-2009/2010 accelerations though the Northeast had the lowest growth. This aligns with the national trends for AF and cirrhosis alone, likely due to regional differences in metabolic risk factors, alcohol consumption, and healthcare access. Our study findings are very similar to the study by Aziz et al, which underscores that dual cardiac-liver disease-related mortality is higher in the South and the West census regions [22].

Current guideline-directed management highlights the complexity of anticoagulation in patients with concomitant cirrhosis and AF. In patients with Child-Pugh A or B cirrhosis and AF, anticoagulation with standard-dose direct oral anticoagulants (DOACs), in accordance with cardiology guideline recommendations for patients without liver disease, is recommended. In those with Child-Pugh C cirrhosis, there is inadequate evidence with respect to the benefit and risk of anticoagulation for stroke prevention in AF [30]. This therapeutic dilemma—balancing stroke prevention against bleeding risk—represents a critical clinical challenge, which may contribute to stroke and stroke-related mortality in AF–cirrhosis; however, studies determining the direct relationship are not sufficient in the literature.

The evolving epidemiology of cirrhosis etiology provides important context for the observed mortality trends. Metabolic dysfunction–associated steatotic liver disease (MASLD) is projected to be a major driver of cirrhosis-related mortality through 2040 [31]. Similarly, mortality from alcohol-associated liver disease (ALD) has nearly doubled in the United States, increasing from 6.71 to 12.53 per 100,000 between 1999 and 2022, with a particularly steep acceleration observed during 2018–2022 (APC = 8.94%) [32]. These transitions, with metabolic and alcohol-related disease replacing viral hepatitis, may contribute to the AF–cirrhosis mortality trends described in our analysis, as both MASLD and ALD carry significant cardiovascular comorbidity burdens [31–33].

Available data also indicate that different liver disease etiologies have distinct relationships with AF. NAFLD/nonalcoholic steatohepatitis (NASH) has been consistently linked to a high risk of incident AF, independent of conventional risk factors, and this association appears stronger in patients with advanced fibrosis [34, 35]. In contrast, chronic viral hepatitis, particularly hepatitis C virus (HCV) infection, has also been associated with elevated AF risk, but this effect seems more modest and partially attenuated by antiviral therapy, suggesting a predominantly inflammatory mechanism [36]. Given that advanced NAFLD/NASH combines cirrhosis-related hemodynamic changes with marked cardiometabolic remodeling, it is biologically plausible that NASH-related cirrhosis may exert a greater impact on atrial structure and function than “lean” viral cirrhosis [37], although direct comparative studies by cirrhosis etiology are currently lacking.

The marked acceleration in AF–cirrhosis-related deaths in recent years, as shown in our study, is consistent with an increased cirrhosis-related mortality from 2016 to 2023. This rise can be primarily attributed to the coronavirus disease 2019 (COVID-19) pandemic’s impact on NAFLD, with additional contributions from disrupted healthcare access and direct COVID-19 effects on patients with chronic liver disease. Before the COVID-19 pandemic, cirrhosis mortality was increasing at a modest rate of approximately 1.95% annually. However, during 2019–2021, this accelerated dramatically to 11.25%. This acceleration was driven primarily by ALD and NAFLD [38]. The pandemic could have also contributed through multiple other mechanisms like social isolation and loss of support systems, increased harmful drinking patterns, and difficulties accessing healthcare services [39].

Lastly, contemporary studies show that consumer-grade and medical-grade wearables detect AF with high sensitivity and specificity and clearly increase AF case ascertainment compared with usual care. This improved detection may therefore partly influence temporal trends in AF-related outcomes [40].

The convergence of the rising trend of cirrhosis and AF–related mortality, etiological transitions behind cirrhosis, and complex anticoagulation strategies highlight multidisciplinary approaches to tackle these issues. In routine practice, cardiology and hepatology often operate in parallel rather than in coordination, which may lead to delayed diagnosis, inconsistent treatment strategies, and uncertainty around anticoagulation. This divide is reflected in the systematic exclusion of cirrhotic patients from major randomized anticoagulation trials, leaving clinicians to rely largely on observational data and expert opinion [14]. Against this backdrop, the accelerating mortality observed in our study, especially among vulnerable groups such as Hispanic individuals and residents of rural areas underscores the urgent need for targeted AF screening, improved access to specialty care, and integrated cardiovascular–hepatology management models. Addressing these gaps will be essential to mitigating the growing public health burden posed by the coexistence of AF and cirrhosis.

This study represents a comprehensive nationwide analysis of age-adjusted mortality trends associated with the coexistence of AF and cirrhosis in the United States using CDC WONDER data; however, several limitations should be acknowledged. First, the analysis relies on death certificate–based cause-of-death coding, which is subject to misclassification and may influence mortality estimates. Additionally, the application of the 2013 National Center for Health Statistics urban–rural classification scheme across all study years may result in misclassification of counties whose urbanization status changed over time, which should be considered when interpreting long-term trends. Furthermore, we were only able to analyze racial disparities for Hispanic, White, and Black or African American patients. Patients of other ethnic groups (Asian or Pacific Islander, American Indian or Alaska Native, etc.) could not be analyzed because of suppressed data for many years in each subgroup. A small proportion of records contained missing demographic or geographic information, limiting the precision of subgroup analyses. Finally, the absence of individual-level clinical and socioeconomic data, including comorbidity burden, disease severity, anticoagulation status, and access to care, precludes adjustment for important confounders and limits causal inference.

### Conclusions

In this nationwide CDC WONDER analysis from 1999 to 2023, mortality associated with the coexistence of cirrhosis and AF increased substantially over time, with age-adjusted mortality rising more than eightfold and accelerating notably after 2016. This increase was observed across all demographic groups, with consistently higher mortality among males, markedly higher rates in adults aged 65 years or older, and the greatest long-term increases among Hispanic or Latino individuals.

Geographic and urbanization-based analyses revealed significant heterogeneity, with the steepest increases in mortality occurring in the West and South, and the most rapid growth observed in noncore rural areas. State level analyses further demonstrated wide variation in AAMRs, indicating that the burden of cirrhosis and AF–related mortality is unevenly distributed across the United States.

Overall, our findings demonstrate a sustained and worsening national mortality trend related to the coexistence of cirrhosis and AF, with accelerating increases in recent years and consistent disparities by age, sex, race and ethnicity, geography, and urban–rural status. These results highlight the growing public health impact of this dual disease burden and underscore the need for focused surveillance and population-level strategies to address the populations and regions experiencing the greatest increases in mortality.

## Supplementary Material

Suppl 1 APC stratified by gender.

Suppl 2 AAPC stratified by gender.

Suppl 3 Age Adjusted Mortality Rate stratified by gender.

Suppl 4 APC stratified by race.

Suppl 5 AAPC stratified by race.

Suppl 6 Age adjusted mortality rate stratified by race.

Suppl 7 APC stratified by age groups.

Suppl 8 Age adjusted mortality rate stratified by age groups.

Suppl 9 APC stratified by census region.

Suppl 10 AAPC stratified by census region.

Suppl 11 Age-adjusted mortality rate stratified by census region.

Suppl 12 APC stratified by urban–rural classification.

Suppl 13 AAPC stratified by urban–rural classification.

Suppl 14 AAMR stratified by urban–rural classification.

Suppl 15 AAMR from 1999 to 2020.

Suppl 16 Total deaths stratified by place of death from 1999 to 2023.

## Data Availability

Any inquiries regarding supporting data availability of this study should be directed to the corresponding author.

## Abbreviations

AF: atrial fibrillation
AAPC: average annual percent change
AAMR: age-adjusted mortality rate
APC: annual percent change
CDC: Centers for Disease Control and Prevention
CDC WONDER: Centers for Disease Control and Prevention Wide-Ranging Online Data for Epidemiologic Research
CI: confidence interval
ICD-10: International Classification of Diseases10th Revision
MASLD: metabolic dysfunction–associated steatotic liver disease
NAFLD: nonalcoholic fatty liver disease
NHW: non-Hispanic White
NHB: non-Hispanic Black
NHAI: non-Hispanic American Indian or Alaska Native
NHA: non-Hispanic Asian or Pacific Islander
RR: relative risk
STROBE: Strengthening the Reporting of Observational Studies in Epidemiology
