# Supplementary material for: Demographic and Geographic Disparities in Atrial Fibrillation and Cirrhosis Mortality in the United States: A Twenty-Five-Year Analysis From 1999 to 2023
Source: Cardiol Res. 2026 Apr 15;17(2):105–19. doi: 10.14740/cr2194 (PMC13094160; doi:10.14740/cr2194)
Supplement: Suppl 1 — APC stratified by gender. [file cr-17-02-105-s001.docx]

**Suppl 1.** APC stratified by gender

| **Sex** | **Years** | **APC (%)** | **95% CI** | **P value** |
| --- | --- | --- | --- | --- |
| Female | 1999–2010 | 3.62 | −17.71 to 38.89 | 0.542 |
| Female | 2010–2013 | 16.58 | −13.23 to 30.00 | 0.503 |
| Female | 2013–2016 | 7.23 | −8.48 to 33.39 | 0.450 |
| Female | 2016–2023 | 14.07 | −14.29 to 44.43 | 0.144 |
| Male | 1999–2003 | 8.89 | 2.32 to 21.89 | 0.012 |
| Male | 2003–2006 | −4.06 | −8.31 to 13.11 | 0.396 |
| Male | 2006–2015 | 9.35 | 4.26 to 18.14 | 0.008 |
| Male | 2015–2023 | 14.77 | 8.00 to 24.46 | 0.018 |
