# Supplementary material for: Demographic and Geographic Disparities in Atrial Fibrillation and Cirrhosis Mortality in the United States: A Twenty-Five-Year Analysis From 1999 to 2023
Source: Cardiol Res. 2026 Apr 15;17(2):105–19. doi: 10.14740/cr2194 (PMC13094160; doi:10.14740/cr2194)
Supplement: Suppl 2 — AAPC stratified by gender. [file cr-17-02-105-s002.docx]

**Suppl 2.** AAPC stratified by gender.

| **Sex** | **Years** | **AAPC (%)** | **95% CI** | **P value** |
| --- | --- | --- | --- | --- |
| Female | 1999–2023 | 8.61 | 6.18 to 11.14 | <0.000001 |
| Male | 1999–2023 | 9.25 | 8.35 to 10.25 | <0.000001 |
