# Supplementary material for: Demographic and Geographic Disparities in Atrial Fibrillation and Cirrhosis Mortality in the United States: A Twenty-Five-Year Analysis From 1999 to 2023
Source: Cardiol Res. 2026 Apr 15;17(2):105–19. doi: 10.14740/cr2194 (PMC13094160; doi:10.14740/cr2194)
Supplement: Suppl 3 — Age Adjusted Mortality Rate stratified by gender. [file cr-17-02-105-s003.docx]

**Suppl 3.** Age Adjusted Mortality Rate stratified by gender.

| Sex | Year | Age Adjusted Rate | Age Adjusted Rate Lower 95% Confidence Interval | Age Adjusted Rate Upper 95% Confidence Interval |
| --- | --- | --- | --- | --- |
| Female | 1999 | 0.2 | 0.1 | 0.2 |
| Female | 2000 | 0.1 | 0.1 | 0.2 |
| Female | 2001 | 0.2 | 0.2 | 0.2 |
| Female | 2002 | 0.2 | 0.1 | 0.2 |
| Female | 2003 | 0.2 | 0.1 | 0.2 |
| Female | 2004 | 0.2 | 0.1 | 0.2 |
| Female | 2005 | 0.2 | 0.2 | 0.2 |
| Female | 2006 | 0.2 | 0.2 | 0.2 |
| Female | 2007 | 0.2 | 0.2 | 0.2 |
| Female | 2008 | 0.2 | 0.2 | 0.3 |
| Female | 2009 | 0.3 | 0.2 | 0.3 |
| Female | 2010 | 0.2 | 0.2 | 0.3 |
| Female | 2011 | 0.3 | 0.3 | 0.3 |
| Female | 2012 | 0.3 | 0.3 | 0.3 |
| Female | 2013 | 0.4 | 0.3 | 0.4 |
| Female | 2014 | 0.4 | 0.3 | 0.4 |
| Female | 2015 | 0.4 | 0.4 | 0.4 |
| Female | 2016 | 0.5 | 0.4 | 0.5 |
| Female | 2017 | 0.5 | 0.5 | 0.6 |
| Female | 2018 | 0.6 | 0.6 | 0.6 |
| Female | 2019 | 0.7 | 0.6 | 0.7 |
| Female | 2020 | 0.8 | 0.8 | 0.9 |
| Female | 2021 | 1 | 0.9 | 1 |
| Female | 2022 | 1 | 1 | 1.1 |
| Female | 2023 | 1.1 | 1 | 1.1 |
| Male | 1999 | 0.3 | 0.3 | 0.4 |
| Male | 2000 | 0.4 | 0.3 | 0.4 |
| Male | 2001 | 0.4 | 0.3 | 0.4 |
| Male | 2002 | 0.4 | 0.3 | 0.4 |
| Male | 2003 | 0.5 | 0.4 | 0.5 |
| Male | 2004 | 0.4 | 0.4 | 0.4 |
| Male | 2005 | 0.4 | 0.4 | 0.5 |
| Male | 2006 | 0.4 | 0.4 | 0.5 |
| Male | 2007 | 0.5 | 0.4 | 0.5 |
| Male | 2008 | 0.5 | 0.4 | 0.5 |
| Male | 2009 | 0.5 | 0.5 | 0.6 |
| Male | 2010 | 0.6 | 0.5 | 0.6 |
| Male | 2011 | 0.6 | 0.6 | 0.7 |
| Male | 2012 | 0.7 | 0.7 | 0.8 |
| Male | 2013 | 0.8 | 0.7 | 0.8 |
| Male | 2014 | 0.8 | 0.8 | 0.9 |
| Male | 2015 | 0.9 | 0.8 | 1 |
| Male | 2016 | 1 | 1 | 1.1 |
| Male | 2017 | 1.2 | 1.1 | 1.2 |
| Male | 2018 | 1.4 | 1.3 | 1.4 |
| Male | 2019 | 1.6 | 1.5 | 1.6 |
| Male | 2020 | 1.9 | 1.8 | 2 |
| Male | 2021 | 2.2 | 2.1 | 2.3 |
| Male | 2022 | 2.4 | 2.3 | 2.5 |
| Male | 2023 | 2.5 | 2.4 | 2.5 |
