# Supplementary material for: Demographic and Geographic Disparities in Atrial Fibrillation and Cirrhosis Mortality in the United States: A Twenty-Five-Year Analysis From 1999 to 2023
Source: Cardiol Res. 2026 Apr 15;17(2):105–19. doi: 10.14740/cr2194 (PMC13094160; doi:10.14740/cr2194)
Supplement: Suppl 4 — APC stratified by race. [file cr-17-02-105-s004.docx]

**Suppl 4.** APC stratified by race.

| **Race / Ethnicity** | **Years** | **APC (%)** | **95% CI** | **P value** |
| --- | --- | --- | --- | --- |
| Black or African American | 1999–2001 | −32.49 | −44.36 to −11.91 | 0.016 |
| Black or African American | 2001–2004 | 23.87 | 6.25 to 39.94 | 0.006 |
| Black or African American | 2004–2011 | 2.22 | −12.36 to 23.57 | 0.916 |
| Black or African American | 2011–2023 | 14.19 | 3.89 to 21.34 | 0.038 |
| White | 1999–2006 | −0.08 | −2.47 to 2.11 | 0.905 |
| White | 2006–2017 | 9.70 | −1.19 to 12.45 | 0.059 |
| White | 2017–2021 | 19.06 | 7.20 to 24.30 | <0.000001 |
| White | 2021–2023 | 7.15 | 1.20 to 16.32 | 0.020 |
| Hispanic or Latino | 2000–2003 | −1.05 | −17.53 to 12.77 | 0.862 |
| Hispanic or Latino | 2003–2007 | 14.67 | −3.11 to 29.99 | 0.114 |
| Hispanic or Latino | 2007–2010 | 2.55 | −4.97 to 26.32 | 0.379 |
| Hispanic or Latino | 2010–2023 | 11.89 | −6.19 to 29.66 | 0.086 |
