# Supplementary material for: Demographic and Geographic Disparities in Atrial Fibrillation and Cirrhosis Mortality in the United States: A Twenty-Five-Year Analysis From 1999 to 2023
Source: Cardiol Res. 2026 Apr 15;17(2):105–19. doi: 10.14740/cr2194 (PMC13094160; doi:10.14740/cr2194)
Supplement: Suppl 5 — AAPC stratified by race. [file cr-17-02-105-s005.docx]

**Suppl 5.** AAPC stratified by race.

| **Race / Ethnicity** | **Years** | **AAPC (%)** | **95% CI** | **P value** |
| --- | --- | --- | --- | --- |
| Black or African American | 1999–2023 | 6.90 | 5.45 to 8.72 | <0.000001 |
| White | 1999–2023 | 8.01 | 7.54 to 8.57 | <0.000001 |
| Hispanic or Latino | 2000–2023 | 9.33 | 7.81 to 10.89 | <0.000001 |
