# Supplementary material for: Demographic and Geographic Disparities in Atrial Fibrillation and Cirrhosis Mortality in the United States: A Twenty-Five-Year Analysis From 1999 to 2023
Source: Cardiol Res. 2026 Apr 15;17(2):105–19. doi: 10.14740/cr2194 (PMC13094160; doi:10.14740/cr2194)
Supplement: Suppl 6 — Age adjusted mortality rate stratified by race. [file cr-17-02-105-s006.docx]

**Suppl 6.** Age adjusted mortality rate stratified by race.

| Race | Year | Age Adjusted Rate | Age Adjusted Rate Lower 95% Confidence Interval | Age Adjusted Rate Upper 95% Confidence Interval |
| --- | --- | --- | --- | --- |
| American Indian or Alaska Native | 2016 | 1.2 | 0.7 | 1.9 |
| American Indian or Alaska Native | 2017 | 1.4 | 0.9 | 2.2 |
| American Indian or Alaska Native | 2018 | 2 | 1.3 | 2.8 |
| American Indian or Alaska Native | 2019 | 1.7 | 1.2 | 2.5 |
| American Indian or Alaska Native | 2020 | 2.4 | 1.8 | 3.3 |
| American Indian or Alaska Native | 2021 | 2.4 | 1.7 | 3.3 |
| American Indian or Alaska Native | 2022 | 1.8 | 1.2 | 2.6 |
| American Indian or Alaska Native | 2023 | 2.4 | 1.7 | 3.2 |
| Asian or Pacific Islander | 2010 | 0.3 | 0.2 | 0.4 |
| Asian or Pacific Islander | 2012 | 0.2 | 0.1 | 0.4 |
| Asian or Pacific Islander | 2013 | 0.2 | 0.1 | 0.4 |
| Asian or Pacific Islander | 2014 | 0.3 | 0.2 | 0.5 |
| Asian or Pacific Islander | 2015 | 0.3 | 0.2 | 0.4 |
| Asian or Pacific Islander | 2016 | 0.3 | 0.2 | 0.4 |
| Asian or Pacific Islander | 2017 | 0.4 | 0.3 | 0.6 |
| Asian or Pacific Islander | 2018 | 0.4 | 0.3 | 0.5 |
| Asian or Pacific Islander | 2019 | 0.5 | 0.4 | 0.6 |
| Asian or Pacific Islander | 2020 | 0.7 | 0.6 | 0.9 |
| Asian or Pacific Islander | 2021 | 0.6 | 0.5 | 0.7 |
| Asian or Pacific Islander | 2022 | 0.6 | 0.5 | 0.7 |
| Asian or Pacific Islander | 2023 | 0.7 | 0.6 | 0.8 |
| Black or African American | 1999 | 0.2 | 0.1 | 0.3 |
| Black or African American | 2000 | 0.2 | 0.1 | 0.2 |
| Black or African American | 2001 | 0.1 | 0.1 | 0.2 |
| Black or African American | 2002 | 0.1 | 0.1 | 0.2 |
| Black or African American | 2003 | 0.2 | 0.1 | 0.2 |
| Black or African American | 2004 | 0.2 | 0.1 | 0.2 |
| Black or African American | 2005 | 0.2 | 0.1 | 0.3 |
| Black or African American | 2006 | 0.2 | 0.1 | 0.2 |
| Black or African American | 2007 | 0.2 | 0.1 | 0.3 |
| Black or African American | 2008 | 0.2 | 0.2 | 0.3 |
| Black or African American | 2009 | 0.2 | 0.1 | 0.3 |
| Black or African American | 2010 | 0.3 | 0.2 | 0.4 |
| Black or African American | 2011 | 0.2 | 0.2 | 0.3 |
| Black or African American | 2012 | 0.3 | 0.2 | 0.4 |
| Black or African American | 2013 | 0.3 | 0.3 | 0.4 |
| Black or African American | 2014 | 0.3 | 0.3 | 0.4 |
| Black or African American | 2015 | 0.4 | 0.3 | 0.5 |
| Black or African American | 2016 | 0.5 | 0.4 | 0.6 |
| Black or African American | 2017 | 0.5 | 0.5 | 0.6 |
| Black or African American | 2018 | 0.5 | 0.4 | 0.6 |
| Black or African American | 2019 | 0.6 | 0.5 | 0.7 |
| Black or African American | 2020 | 0.9 | 0.8 | 1 |
| Black or African American | 2021 | 0.9 | 0.8 | 1.1 |
| Black or African American | 2022 | 1 | 0.9 | 1.1 |
| Black or African American | 2023 | 1.1 | 1 | 1.2 |
| White | 1999 | 0.3 | 0.2 | 0.3 |
| White | 2000 | 0.3 | 0.2 | 0.3 |
| White | 2001 | 0.3 | 0.3 | 0.3 |
| White | 2002 | 0.3 | 0.3 | 0.3 |
| White | 2003 | 0.3 | 0.3 | 0.4 |
| White | 2004 | 0.3 | 0.3 | 0.3 |
| White | 2005 | 0.3 | 0.3 | 0.3 |
| White | 2006 | 0.3 | 0.3 | 0.4 |
| White | 2007 | 0.3 | 0.3 | 0.4 |
| White | 2008 | 0.4 | 0.3 | 0.4 |
| White | 2009 | 0.4 | 0.3 | 0.4 |
| White | 2010 | 0.4 | 0.4 | 0.4 |
| White | 2011 | 0.5 | 0.4 | 0.5 |
| White | 2012 | 0.5 | 0.5 | 0.6 |
| White | 2013 | 0.6 | 0.5 | 0.6 |
| White | 2014 | 0.6 | 0.5 | 0.6 |
| White | 2015 | 0.7 | 0.6 | 0.7 |
| White | 2016 | 0.8 | 0.7 | 0.8 |
| White | 2017 | 0.8 | 0.8 | 0.9 |
| White | 2018 | 1 | 1 | 1.1 |
| White | 2019 | 1.1 | 1.1 | 1.2 |
| White | 2020 | 1.4 | 1.3 | 1.4 |
| White | 2021 | 1.7 | 1.6 | 1.7 |
| White | 2022 | 1.8 | 1.8 | 1.9 |
| White | 2023 | 1.9 | 1.8 | 1.9 |
| Hispanic or Latino | 2000 | 0.2 | 0.1 | 0.3 |
| Hispanic or Latino | 2001 | 0.3 | 0.2 | 0.4 |
| Hispanic or Latino | 2002 | 0.2 | 0.1 | 0.4 |
| Hispanic or Latino | 2003 | 0.2 | 0.1 | 0.3 |
| Hispanic or Latino | 2004 | 0.3 | 0.2 | 0.4 |
| Hispanic or Latino | 2005 | 0.3 | 0.2 | 0.4 |
| Hispanic or Latino | 2006 | 0.3 | 0.2 | 0.4 |
| Hispanic or Latino | 2007 | 0.4 | 0.3 | 0.5 |
| Hispanic or Latino | 2008 | 0.4 | 0.3 | 0.5 |
| Hispanic or Latino | 2009 | 0.4 | 0.3 | 0.5 |
| Hispanic or Latino | 2010 | 0.4 | 0.3 | 0.5 |
| Hispanic or Latino | 2011 | 0.5 | 0.4 | 0.6 |
| Hispanic or Latino | 2012 | 0.5 | 0.4 | 0.7 |
| Hispanic or Latino | 2013 | 0.6 | 0.5 | 0.7 |
| Hispanic or Latino | 2014 | 0.6 | 0.5 | 0.7 |
| Hispanic or Latino | 2015 | 0.7 | 0.6 | 0.8 |
| Hispanic or Latino | 2016 | 0.9 | 0.7 | 1 |
| Hispanic or Latino | 2017 | 1 | 0.9 | 1.2 |
| Hispanic or Latino | 2018 | 0.9 | 0.8 | 1 |
| Hispanic or Latino | 2019 | 1.2 | 1 | 1.3 |
| Hispanic or Latino | 2020 | 1.4 | 1.2 | 1.5 |
| Hispanic or Latino | 2021 | 1.5 | 1.3 | 1.6 |
| Hispanic or Latino | 2022 | 1.5 | 1.3 | 1.6 |
| Hispanic or Latino | 2023 | 1.7 | 1.5 | 1.8 |
