# Supplementary material for: Demographic and Geographic Disparities in Atrial Fibrillation and Cirrhosis Mortality in the United States: A Twenty-Five-Year Analysis From 1999 to 2023
Source: Cardiol Res. 2026 Apr 15;17(2):105–19. doi: 10.14740/cr2194 (PMC13094160; doi:10.14740/cr2194)
Supplement: Suppl 7 — APC stratified by age groups. [file cr-17-02-105-s007.docx]

**Suppl 7.** APC stratified by age groups.

| **Age Group** | **Years** | **APC (%)** | **95% CI** | **P value** |
| --- | --- | --- | --- | --- |
| ≥65 years | 1999–2010 | 3.84 | 0.69 to 5.63 | 0.028 |
| ≥65 years | 2010–2023 | 12.61 | 11.13 to 14.99 | <0.000001 |
| 25–64 years | 1999–2011 | 0.02 | −2.37 to 1.83 | 0.989 |
| 25–64 years | 2011–2023 | 13.38 | 11.31 to 16.03 | <0.000001 |
