# Supplementary material for: Demographic and Geographic Disparities in Atrial Fibrillation and Cirrhosis Mortality in the United States: A Twenty-Five-Year Analysis From 1999 to 2023
Source: Cardiol Res. 2026 Apr 15;17(2):105–19. doi: 10.14740/cr2194 (PMC13094160; doi:10.14740/cr2194)
Supplement: Suppl 8 — Age adjusted mortality rate stratified by age groups. [file cr-17-02-105-s008.docx]

**Suppl 8.** Age adjusted mortality rate stratified by age groups.

| Age groups | Year | Age Adjusted Rate | Age Adjusted Rate Lower 95% Confidence Interval | Age Adjusted Rate Upper 95% Confidence Interval |
| --- | --- | --- | --- | --- |
| Greater than or equal to 65 years | 1999 | 1 | 0.9 | 1.1 |
| Greater than or equal to 65 years | 2000 | 1 | 0.9 | 1.1 |
| Greater than or equal to 65 years | 2001 | 1.2 | 1.1 | 1.3 |
| Greater than or equal to 65 years | 2002 | 1.1 | 1 | 1.2 |
| Greater than or equal to 65 years | 2003 | 1.3 | 1.1 | 1.4 |
| Greater than or equal to 65 years | 2004 | 1.1 | 1 | 1.2 |
| Greater than or equal to 65 years | 2005 | 1.2 | 1.1 | 1.3 |
| Greater than or equal to 65 years | 2006 | 1.3 | 1.2 | 1.4 |
| Greater than or equal to 65 years | 2007 | 1.3 | 1.2 | 1.5 |
| Greater than or equal to 65 years | 2008 | 1.5 | 1.4 | 1.6 |
| Greater than or equal to 65 years | 2009 | 1.5 | 1.3 | 1.6 |
| Greater than or equal to 65 years | 2010 | 1.6 | 1.5 | 1.8 |
| Greater than or equal to 65 years | 2011 | 1.8 | 1.7 | 2 |
| Greater than or equal to 65 years | 2012 | 2 | 1.8 | 2.1 |
| Greater than or equal to 65 years | 2013 | 2.3 | 2.2 | 2.5 |
| Greater than or equal to 65 years | 2014 | 2.3 | 2.1 | 2.4 |
| Greater than or equal to 65 years | 2015 | 2.5 | 2.3 | 2.6 |
| Greater than or equal to 65 years | 2016 | 2.9 | 2.8 | 3.1 |
| Greater than or equal to 65 years | 2017 | 3.3 | 3.2 | 3.5 |
| Greater than or equal to 65 years | 2018 | 3.9 | 3.7 | 4 |
| Greater than or equal to 65 years | 2019 | 4.3 | 4.1 | 4.5 |
| Greater than or equal to 65 years | 2020 | 5.2 | 5 | 5.4 |
| Greater than or equal to 65 years | 2021 | 6 | 5.8 | 6.2 |
| Greater than or equal to 65 years | 2022 | 6.7 | 6.4 | 6.9 |
| Greater than or equal to 65 years | 2023 | 7.1 | 6.8 | 7.3 |
| From 25-64 years old | 1999 | 0.1 | 0 | 0.1 |
| From 25-64 years old | 2000 | 0.1 | 0 | 0.1 |
| From 25-64 years old | 2001 | 0.1 | 0 | 0.1 |
| From 25-64 years old | 2002 | 0.1 | 0 | 0.1 |
| From 25-64 years old | 2003 | 0.1 | 0.1 | 0.1 |
| From 25-64 years old | 2004 | 0.1 | 0.1 | 0.1 |
| From 25-64 years old | 2005 | 0.1 | 0 | 0.1 |
| From 25-64 years old | 2006 | 0.1 | 0 | 0.1 |
| From 25-64 years old | 2007 | 0.1 | 0.1 | 0.1 |
| From 25-64 years old | 2008 | 0.1 | 0.1 | 0.1 |
| From 25-64 years old | 2009 | 0.1 | 0.1 | 0.1 |
| From 25-64 years old | 2010 | 0.1 | 0.1 | 0.1 |
| From 25-64 years old | 2011 | 0.1 | 0.1 | 0.1 |
| From 25-64 years old | 2012 | 0.1 | 0.1 | 0.1 |
| From 25-64 years old | 2013 | 0.1 | 0.1 | 0.1 |
| From 25-64 years old | 2014 | 0.2 | 0.1 | 0.2 |
| From 25-64 years old | 2015 | 0.2 | 0.2 | 0.2 |
| From 25-64 years old | 2016 | 0.2 | 0.2 | 0.2 |
| From 25-64 years old | 2017 | 0.2 | 0.2 | 0.2 |
| From 25-64 years old | 2018 | 0.2 | 0.2 | 0.3 |
| From 25-64 years old | 2019 | 0.3 | 0.2 | 0.3 |
| From 25-64 years old | 2020 | 0.3 | 0.3 | 0.4 |
| From 25-64 years old | 2021 | 0.4 | 0.4 | 0.5 |
| From 25-64 years old | 2022 | 0.4 | 0.4 | 0.4 |
| From 25-64 years old | 2023 | 0.4 | 0.4 | 0.4 |
