# Supplementary material for: Demographic and Geographic Disparities in Atrial Fibrillation and Cirrhosis Mortality in the United States: A Twenty-Five-Year Analysis From 1999 to 2023
Source: Cardiol Res. 2026 Apr 15;17(2):105–19. doi: 10.14740/cr2194 (PMC13094160; doi:10.14740/cr2194)
Supplement: Suppl 9 — APC stratified by census region. [file cr-17-02-105-s009.docx]

**Suppl 9.** APC stratified by Census region.

| **U.S. Census Region** | **Years** | **APC (%)** | **95% CI** | **P value** |
| --- | --- | --- | --- | --- |
| Northeast | 1999–2009 | 1.47 | −5.09 to 4.34 | 0.476 |
| Northeast | 2009–2023 | 10.02 | 8.14 to 13.70 | <0.000001 |
| Midwest | 1999–2015 | 6.30 | 4.08 to 7.67 | 0.0036 |
| Midwest | 2015–2023 | 15.87 | 11.85 to 24.98 | <0.000001 |
| South | 1999–2009 | 4.19 | −1.06 to 6.82 | 0.0788 |
| South | 2009–2023 | 13.41 | 11.66 to 16.23 | <0.000001 |
| West | 1999–2012 | 7.58 | 0.33 to 9.46 | 0.0468 |
| West | 2012–2023 | 13.43 | 10.94 to 23.63 | 0.0032 |
