# Supplementary material for: Demographic and Geographic Disparities in Atrial Fibrillation and Cirrhosis Mortality in the United States: A Twenty-Five-Year Analysis From 1999 to 2023
Source: Cardiol Res. 2026 Apr 15;17(2):105–19. doi: 10.14740/cr2194 (PMC13094160; doi:10.14740/cr2194)
Supplement: Suppl 10 — AAPC stratified by census region. [file cr-17-02-105-s010.docx]

**Suppl 10.** AAPC stratified by Census region.

| **U.S. Census Region** | **Years** | **AAPC (%)** | **95% CI** | **P value** |
| --- | --- | --- | --- | --- |
| Northeast | 1999–2023 | 6.37 | 5.23 to 7.48 | <0.000001 |
| Midwest | 1999–2023 | 9.40 | 8.35 to 10.38 | <0.000001 |
| South | 1999–2023 | 9.47 | 8.51 to 10.45 | <0.000001 |
| West | 1999–2023 | 10.22 | 9.04 to 11.33 | <0.000001 |
