# Supplementary material for: Demographic and Geographic Disparities in Atrial Fibrillation and Cirrhosis Mortality in the United States: A Twenty-Five-Year Analysis From 1999 to 2023
Source: Cardiol Res. 2026 Apr 15;17(2):105–19. doi: 10.14740/cr2194 (PMC13094160; doi:10.14740/cr2194)
Supplement: Suppl 11 — Age-adjusted mortality rate stratified by census region. [file cr-17-02-105-s011.docx]

**Suppl 11.** Age-adjusted mortality rate stratified by Census region.

| Census Region | Year | Age Adjusted Rate | Age Adjusted Rate Lower 95% Confidence Interval | Age Adjusted Rate Upper 95% Confidence Interval |
| --- | --- | --- | --- | --- |
| Census Region 1: Northeast | 1999 | 0.2 | 0.2 | 0.3 |
| Census Region 1: Northeast | 2000 | 0.3 | 0.2 | 0.3 |
| Census Region 1: Northeast | 2001 | 0.3 | 0.2 | 0.3 |
| Census Region 1: Northeast | 2002 | 0.3 | 0.3 | 0.4 |
| Census Region 1: Northeast | 2003 | 0.4 | 0.3 | 0.4 |
| Census Region 1: Northeast | 2004 | 0.3 | 0.2 | 0.3 |
| Census Region 1: Northeast | 2005 | 0.3 | 0.3 | 0.4 |
| Census Region 1: Northeast | 2006 | 0.3 | 0.2 | 0.3 |
| Census Region 1: Northeast | 2007 | 0.3 | 0.3 | 0.4 |
| Census Region 1: Northeast | 2008 | 0.3 | 0.3 | 0.4 |
| Census Region 1: Northeast | 2009 | 0.3 | 0.3 | 0.4 |
| Census Region 1: Northeast | 2010 | 0.4 | 0.4 | 0.5 |
| Census Region 1: Northeast | 2011 | 0.4 | 0.4 | 0.5 |
| Census Region 1: Northeast | 2012 | 0.4 | 0.4 | 0.5 |
| Census Region 1: Northeast | 2013 | 0.5 | 0.4 | 0.6 |
| Census Region 1: Northeast | 2014 | 0.5 | 0.4 | 0.5 |
| Census Region 1: Northeast | 2015 | 0.5 | 0.5 | 0.6 |
| Census Region 1: Northeast | 2016 | 0.6 | 0.5 | 0.7 |
| Census Region 1: Northeast | 2017 | 0.6 | 0.5 | 0.6 |
| Census Region 1: Northeast | 2018 | 0.7 | 0.6 | 0.8 |
| Census Region 1: Northeast | 2019 | 0.7 | 0.7 | 0.8 |
| Census Region 1: Northeast | 2020 | 1.1 | 1 | 1.1 |
| Census Region 1: Northeast | 2021 | 1 | 0.9 | 1.1 |
| Census Region 1: Northeast | 2022 | 1.2 | 1.1 | 1.3 |
| Census Region 1: Northeast | 2023 | 1.3 | 1.2 | 1.4 |
| Census Region 2: Midwest | 1999 | 0.2 | 0.2 | 0.3 |
| Census Region 2: Midwest | 2000 | 0.2 | 0.2 | 0.3 |
| Census Region 2: Midwest | 2001 | 0.2 | 0.2 | 0.3 |
| Census Region 2: Midwest | 2002 | 0.3 | 0.2 | 0.3 |
| Census Region 2: Midwest | 2003 | 0.3 | 0.2 | 0.3 |
| Census Region 2: Midwest | 2004 | 0.2 | 0.2 | 0.3 |
| Census Region 2: Midwest | 2005 | 0.3 | 0.2 | 0.3 |
| Census Region 2: Midwest | 2006 | 0.3 | 0.3 | 0.4 |
| Census Region 2: Midwest | 2007 | 0.3 | 0.3 | 0.4 |
| Census Region 2: Midwest | 2008 | 0.3 | 0.3 | 0.4 |
| Census Region 2: Midwest | 2009 | 0.4 | 0.3 | 0.4 |
| Census Region 2: Midwest | 2010 | 0.4 | 0.3 | 0.4 |
| Census Region 2: Midwest | 2011 | 0.4 | 0.3 | 0.4 |
| Census Region 2: Midwest | 2012 | 0.4 | 0.4 | 0.5 |
| Census Region 2: Midwest | 2013 | 0.5 | 0.4 | 0.5 |
| Census Region 2: Midwest | 2014 | 0.5 | 0.4 | 0.6 |
| Census Region 2: Midwest | 2015 | 0.5 | 0.5 | 0.6 |
| Census Region 2: Midwest | 2016 | 0.6 | 0.5 | 0.7 |
| Census Region 2: Midwest | 2017 | 0.7 | 0.6 | 0.8 |
| Census Region 2: Midwest | 2018 | 0.8 | 0.7 | 0.9 |
| Census Region 2: Midwest | 2019 | 1 | 0.9 | 1 |
| Census Region 2: Midwest | 2020 | 1.2 | 1.1 | 1.3 |
| Census Region 2: Midwest | 2021 | 1.3 | 1.2 | 1.4 |
| Census Region 2: Midwest | 2022 | 1.5 | 1.4 | 1.6 |
| Census Region 2: Midwest | 2023 | 1.5 | 1.4 | 1.6 |
| Census Region 3: South | 1999 | 0.2 | 0.2 | 0.3 |
| Census Region 3: South | 2000 | 0.2 | 0.2 | 0.3 |
| Census Region 3: South | 2001 | 0.3 | 0.2 | 0.3 |
| Census Region 3: South | 2002 | 0.2 | 0.2 | 0.3 |
| Census Region 3: South | 2003 | 0.3 | 0.2 | 0.3 |
| Census Region 3: South | 2004 | 0.3 | 0.2 | 0.3 |
| Census Region 3: South | 2005 | 0.3 | 0.2 | 0.3 |
| Census Region 3: South | 2006 | 0.3 | 0.2 | 0.3 |
| Census Region 3: South | 2007 | 0.3 | 0.3 | 0.3 |
| Census Region 3: South | 2008 | 0.3 | 0.3 | 0.4 |
| Census Region 3: South | 2009 | 0.3 | 0.3 | 0.4 |
| Census Region 3: South | 2010 | 0.4 | 0.3 | 0.4 |
| Census Region 3: South | 2011 | 0.4 | 0.4 | 0.5 |
| Census Region 3: South | 2012 | 0.5 | 0.5 | 0.6 |
| Census Region 3: South | 2013 | 0.6 | 0.5 | 0.6 |
| Census Region 3: South | 2014 | 0.6 | 0.5 | 0.6 |
| Census Region 3: South | 2015 | 0.6 | 0.6 | 0.7 |
| Census Region 3: South | 2016 | 0.8 | 0.7 | 0.8 |
| Census Region 3: South | 2017 | 0.9 | 0.9 | 1 |
| Census Region 3: South | 2018 | 1 | 1 | 1.1 |
| Census Region 3: South | 2019 | 1.2 | 1.1 | 1.3 |
| Census Region 3: South | 2020 | 1.3 | 1.3 | 1.4 |
| Census Region 3: South | 2021 | 1.6 | 1.5 | 1.7 |
| Census Region 3: South | 2022 | 1.8 | 1.7 | 1.9 |
| Census Region 3: South | 2023 | 1.8 | 1.8 | 1.9 |
| Census Region 4: West | 1999 | 0.2 | 0.2 | 0.3 |
| Census Region 4: West | 2000 | 0.2 | 0.2 | 0.3 |
| Census Region 4: West | 2001 | 0.3 | 0.2 | 0.3 |
| Census Region 4: West | 2002 | 0.2 | 0.2 | 0.3 |
| Census Region 4: West | 2003 | 0.3 | 0.3 | 0.4 |
| Census Region 4: West | 2004 | 0.3 | 0.3 | 0.4 |
| Census Region 4: West | 2005 | 0.3 | 0.3 | 0.4 |
| Census Region 4: West | 2006 | 0.4 | 0.3 | 0.4 |
| Census Region 4: West | 2007 | 0.4 | 0.3 | 0.5 |
| Census Region 4: West | 2008 | 0.5 | 0.4 | 0.5 |
| Census Region 4: West | 2009 | 0.4 | 0.3 | 0.5 |
| Census Region 4: West | 2010 | 0.4 | 0.4 | 0.5 |
| Census Region 4: West | 2011 | 0.5 | 0.4 | 0.6 |
| Census Region 4: West | 2012 | 0.5 | 0.5 | 0.6 |
| Census Region 4: West | 2013 | 0.6 | 0.6 | 0.7 |
| Census Region 4: West | 2014 | 0.7 | 0.6 | 0.8 |
| Census Region 4: West | 2015 | 0.8 | 0.7 | 0.8 |
| Census Region 4: West | 2016 | 0.9 | 0.8 | 1 |
| Census Region 4: West | 2017 | 1 | 0.9 | 1.1 |
| Census Region 4: West | 2018 | 1.1 | 1 | 1.2 |
| Census Region 4: West | 2019 | 1.2 | 1.2 | 1.3 |
| Census Region 4: West | 2020 | 1.5 | 1.4 | 1.6 |
| Census Region 4: West | 2021 | 1.9 | 1.8 | 2 |
| Census Region 4: West | 2022 | 1.9 | 1.8 | 2 |
| Census Region 4: West | 2023 | 2 | 1.9 | 2.2 |
