# Supplementary material for: Demographic and Geographic Disparities in Atrial Fibrillation and Cirrhosis Mortality in the United States: A Twenty-Five-Year Analysis From 1999 to 2023
Source: Cardiol Res. 2026 Apr 15;17(2):105–19. doi: 10.14740/cr2194 (PMC13094160; doi:10.14740/cr2194)
Supplement: Suppl 12 — APC stratified by urban–rural classification. [file cr-17-02-105-s012.docx]

**Suppl 12.** APC stratified by urban-rural classification.

| **Urban–Rural Classification** | **Years** | **APC (%)** | **95% CI** | **P value** |
| --- | --- | --- | --- | --- |
| Large Central Metro | 1999–2012 | 5.88 | 2.51 to 7.40 | 0.020 |
| Large Central Metro | 2012–2020 | 13.48 | 10.27 to 21.96 | <0.000001 |
| Large Fringe Metro | 1999–2009 | 2.20 | −3.75 to 4.82 | 0.287 |
| Large Fringe Metro | 2009–2020 | 10.52 | 8.21 to 16.25 | 0.0008 |
| Medium Metro | 1999–2010 | 4.20 | −0.48 to 6.54 | 0.066 |
| Medium Metro | 2010–2020 | 13.19 | 10.34 to 19.26 | <0.000001 |
| Small Metro | 1999–2007 | 2.91 | −15.56 to 8.18 | 0.611 |
| Small Metro | 2007–2020 | 11.69 | 8.95 to 29.53 | 0.015 |
| Micropolitan (Nonmetro) | 1999–2004 | −2.73 | −24.41 to 9.35 | 0.677 |
| Micropolitan (Nonmetro) | 2004–2020 | 11.10 | 7.93 to 27.27 | 0.039 |
| Noncore (Nonmetro) | 1999–2010 | 4.48 | 0.73 to 6.77 | 0.030 |
| Noncore (Nonmetro) | 2010–2020 | 16.25 | 13.25 to 21.27 | <0.000001 |
