# Supplementary material for: Demographic and Geographic Disparities in Atrial Fibrillation and Cirrhosis Mortality in the United States: A Twenty-Five-Year Analysis From 1999 to 2023
Source: Cardiol Res. 2026 Apr 15;17(2):105–19. doi: 10.14740/cr2194 (PMC13094160; doi:10.14740/cr2194)
Supplement: Suppl 13 — AAPC stratified by urban–rural classification. [file cr-17-02-105-s013.docx]

**Suppl 13.** AAPC stratified by urban-rural classification.

| **Urban–Rural Classification** | **Years** | **AAPC (%)** | **95% CI** | **P value** |
| --- | --- | --- | --- | --- |
| Large Central Metro | 1999–2020 | 8.71 | 7.69 to 9.70 | <0.000001 |
| Large Fringe Metro | 1999–2020 | 6.48 | 5.36 to 7.67 | <0.000001 |
| Medium Metro | 1999–2020 | 8.39 | 7.14 to 9.63 | <0.000001 |
| Small Metro | 1999–2020 | 8.26 | 6.28 to 10.47 | <0.000001 |
| Micropolitan (Nonmetro) | 1999–2020 | 7.64 | 5.59 to 10.62 | <0.000001 |
| Noncore (Nonmetro) | 1999–2020 | 9.93 | 8.71 to 11.18 | <0.000001 |
