# Supplementary material for: Demographic and Geographic Disparities in Atrial Fibrillation and Cirrhosis Mortality in the United States: A Twenty-Five-Year Analysis From 1999 to 2023
Source: Cardiol Res. 2026 Apr 15;17(2):105–19. doi: 10.14740/cr2194 (PMC13094160; doi:10.14740/cr2194)
Supplement: Suppl 14 — AAMR stratified by urban–rural classification. [file cr-17-02-105-s014.docx]

**Suppl 14.** AAMR stratified by urban rural classification.

| 2013 Urbanization | Year | Age Adjusted Rate | Age Adjusted Rate Lower 95% Confidence Interval | Age Adjusted Rate Upper 95% Confidence Interval |
| --- | --- | --- | --- | --- |
| Large Central Metro | 1999 | 0.2 | 0.2 | 0.3 |
| Large Central Metro | 2000 | 0.2 | 0.2 | 0.2 |
| Large Central Metro | 2001 | 0.2 | 0.2 | 0.3 |
| Large Central Metro | 2002 | 0.2 | 0.2 | 0.3 |
| Large Central Metro | 2003 | 0.3 | 0.2 | 0.3 |
| Large Central Metro | 2004 | 0.3 | 0.2 | 0.3 |
| Large Central Metro | 2005 | 0.3 | 0.2 | 0.3 |
| Large Central Metro | 2006 | 0.3 | 0.3 | 0.3 |
| Large Central Metro | 2007 | 0.3 | 0.3 | 0.4 |
| Large Central Metro | 2008 | 0.3 | 0.3 | 0.4 |
| Large Central Metro | 2009 | 0.3 | 0.3 | 0.4 |
| Large Central Metro | 2010 | 0.4 | 0.4 | 0.5 |
| Large Central Metro | 2011 | 0.4 | 0.3 | 0.4 |
| Large Central Metro | 2012 | 0.4 | 0.4 | 0.5 |
| Large Central Metro | 2013 | 0.5 | 0.5 | 0.6 |
| Large Central Metro | 2014 | 0.5 | 0.5 | 0.6 |
| Large Central Metro | 2015 | 0.6 | 0.5 | 0.7 |
| Large Central Metro | 2016 | 0.7 | 0.6 | 0.8 |
| Large Central Metro | 2017 | 0.8 | 0.7 | 0.8 |
| Large Central Metro | 2018 | 0.9 | 0.8 | 0.9 |
| Large Central Metro | 2019 | 0.9 | 0.9 | 1 |
| Large Central Metro | 2020 | 1.2 | 1.1 | 1.2 |
| Large Fringe Metro | 1999 | 0.2 | 0.2 | 0.3 |
| Large Fringe Metro | 2000 | 0.3 | 0.2 | 0.3 |
| Large Fringe Metro | 2001 | 0.3 | 0.2 | 0.3 |
| Large Fringe Metro | 2002 | 0.3 | 0.2 | 0.3 |
| Large Fringe Metro | 2003 | 0.3 | 0.2 | 0.3 |
| Large Fringe Metro | 2004 | 0.3 | 0.2 | 0.3 |
| Large Fringe Metro | 2005 | 0.3 | 0.2 | 0.3 |
| Large Fringe Metro | 2006 | 0.3 | 0.2 | 0.3 |
| Large Fringe Metro | 2007 | 0.3 | 0.3 | 0.4 |
| Large Fringe Metro | 2008 | 0.3 | 0.3 | 0.4 |
| Large Fringe Metro | 2009 | 0.3 | 0.3 | 0.4 |
| Large Fringe Metro | 2010 | 0.4 | 0.3 | 0.4 |
| Large Fringe Metro | 2011 | 0.4 | 0.4 | 0.5 |
| Large Fringe Metro | 2012 | 0.5 | 0.4 | 0.5 |
| Large Fringe Metro | 2013 | 0.5 | 0.4 | 0.5 |
| Large Fringe Metro | 2014 | 0.5 | 0.4 | 0.6 |
| Large Fringe Metro | 2015 | 0.5 | 0.5 | 0.6 |
| Large Fringe Metro | 2016 | 0.6 | 0.6 | 0.7 |
| Large Fringe Metro | 2017 | 0.7 | 0.6 | 0.7 |
| Large Fringe Metro | 2018 | 0.8 | 0.7 | 0.8 |
| Large Fringe Metro | 2019 | 0.9 | 0.8 | 0.9 |
| Large Fringe Metro | 2020 | 1.1 | 1 | 1.2 |
| Medium Metro | 1999 | 0.2 | 0.2 | 0.3 |
| Medium Metro | 2000 | 0.3 | 0.2 | 0.3 |
| Medium Metro | 2001 | 0.3 | 0.2 | 0.4 |
| Medium Metro | 2002 | 0.3 | 0.3 | 0.4 |
| Medium Metro | 2003 | 0.4 | 0.3 | 0.4 |
| Medium Metro | 2004 | 0.3 | 0.2 | 0.3 |
| Medium Metro | 2005 | 0.3 | 0.3 | 0.4 |
| Medium Metro | 2006 | 0.4 | 0.3 | 0.4 |
| Medium Metro | 2007 | 0.3 | 0.3 | 0.4 |
| Medium Metro | 2008 | 0.4 | 0.3 | 0.4 |
| Medium Metro | 2009 | 0.4 | 0.4 | 0.5 |
| Medium Metro | 2010 | 0.4 | 0.4 | 0.5 |
| Medium Metro | 2011 | 0.4 | 0.4 | 0.5 |
| Medium Metro | 2012 | 0.6 | 0.5 | 0.6 |
| Medium Metro | 2013 | 0.6 | 0.5 | 0.7 |
| Medium Metro | 2014 | 0.7 | 0.6 | 0.7 |
| Medium Metro | 2015 | 0.7 | 0.6 | 0.7 |
| Medium Metro | 2016 | 0.8 | 0.7 | 0.9 |
| Medium Metro | 2017 | 1 | 0.9 | 1.1 |
| Medium Metro | 2018 | 1.1 | 1 | 1.2 |
| Medium Metro | 2019 | 1.3 | 1.2 | 1.4 |
| Medium Metro | 2020 | 1.4 | 1.3 | 1.5 |
| Small Metro | 1999 | 0.3 | 0.2 | 0.3 |
| Small Metro | 2000 | 0.2 | 0.2 | 0.3 |
| Small Metro | 2001 | 0.3 | 0.2 | 0.4 |
| Small Metro | 2002 | 0.2 | 0.2 | 0.3 |
| Small Metro | 2003 | 0.3 | 0.2 | 0.4 |
| Small Metro | 2004 | 0.3 | 0.2 | 0.4 |
| Small Metro | 2005 | 0.3 | 0.3 | 0.4 |
| Small Metro | 2006 | 0.3 | 0.2 | 0.4 |
| Small Metro | 2007 | 0.2 | 0.2 | 0.3 |
| Small Metro | 2008 | 0.5 | 0.4 | 0.6 |
| Small Metro | 2009 | 0.4 | 0.3 | 0.5 |
| Small Metro | 2010 | 0.5 | 0.4 | 0.5 |
| Small Metro | 2011 | 0.6 | 0.5 | 0.7 |
| Small Metro | 2012 | 0.5 | 0.4 | 0.6 |
| Small Metro | 2013 | 0.6 | 0.5 | 0.7 |
| Small Metro | 2014 | 0.5 | 0.4 | 0.6 |
| Small Metro | 2015 | 0.7 | 0.6 | 0.8 |
| Small Metro | 2016 | 0.8 | 0.7 | 0.9 |
| Small Metro | 2017 | 0.9 | 0.8 | 1 |
| Small Metro | 2018 | 1.1 | 0.9 | 1.2 |
| Small Metro | 2019 | 1.1 | 1 | 1.3 |
| Small Metro | 2020 | 1.5 | 1.3 | 1.6 |
| Micropolitan (Nonmetro) | 1999 | 0.3 | 0.2 | 0.3 |
| Micropolitan (Nonmetro) | 2000 | 0.2 | 0.1 | 0.3 |
| Micropolitan (Nonmetro) | 2001 | 0.3 | 0.2 | 0.3 |
| Micropolitan (Nonmetro) | 2002 | 0.3 | 0.2 | 0.3 |
| Micropolitan (Nonmetro) | 2003 | 0.3 | 0.2 | 0.4 |
| Micropolitan (Nonmetro) | 2004 | 0.2 | 0.2 | 0.3 |
| Micropolitan (Nonmetro) | 2005 | 0.3 | 0.2 | 0.4 |
| Micropolitan (Nonmetro) | 2006 | 0.3 | 0.2 | 0.4 |
| Micropolitan (Nonmetro) | 2007 | 0.4 | 0.3 | 0.5 |
| Micropolitan (Nonmetro) | 2008 | 0.3 | 0.3 | 0.4 |
| Micropolitan (Nonmetro) | 2009 | 0.5 | 0.4 | 0.6 |
| Micropolitan (Nonmetro) | 2010 | 0.4 | 0.4 | 0.5 |
| Micropolitan (Nonmetro) | 2011 | 0.5 | 0.4 | 0.6 |
| Micropolitan (Nonmetro) | 2012 | 0.5 | 0.4 | 0.6 |
| Micropolitan (Nonmetro) | 2013 | 0.7 | 0.6 | 0.8 |
| Micropolitan (Nonmetro) | 2014 | 0.6 | 0.5 | 0.7 |
| Micropolitan (Nonmetro) | 2015 | 0.7 | 0.6 | 0.9 |
| Micropolitan (Nonmetro) | 2016 | 0.7 | 0.6 | 0.9 |
| Micropolitan (Nonmetro) | 2017 | 0.8 | 0.7 | 0.9 |
| Micropolitan (Nonmetro) | 2018 | 1.2 | 1.1 | 1.3 |
| Micropolitan (Nonmetro) | 2019 | 1.4 | 1.2 | 1.5 |
| Micropolitan (Nonmetro) | 2020 | 1.6 | 1.4 | 1.7 |
| Noncore (Nonmetro) | 1999 | 0.2 | 0.2 | 0.3 |
| Noncore (Nonmetro) | 2000 | 0.2 | 0.1 | 0.3 |
| Noncore (Nonmetro) | 2001 | 0.3 | 0.2 | 0.4 |
| Noncore (Nonmetro) | 2002 | 0.2 | 0.1 | 0.3 |
| Noncore (Nonmetro) | 2003 | 0.2 | 0.2 | 0.3 |
| Noncore (Nonmetro) | 2004 | 0.2 | 0.1 | 0.3 |
| Noncore (Nonmetro) | 2005 | 0.3 | 0.2 | 0.4 |
| Noncore (Nonmetro) | 2006 | 0.3 | 0.2 | 0.4 |
| Noncore (Nonmetro) | 2007 | 0.3 | 0.2 | 0.4 |
| Noncore (Nonmetro) | 2008 | 0.3 | 0.2 | 0.4 |
| Noncore (Nonmetro) | 2009 | 0.3 | 0.2 | 0.4 |
| Noncore (Nonmetro) | 2010 | 0.3 | 0.3 | 0.5 |
| Noncore (Nonmetro) | 2011 | 0.4 | 0.3 | 0.5 |
| Noncore (Nonmetro) | 2012 | 0.5 | 0.4 | 0.6 |
| Noncore (Nonmetro) | 2013 | 0.5 | 0.4 | 0.6 |
| Noncore (Nonmetro) | 2014 | 0.6 | 0.5 | 0.7 |
| Noncore (Nonmetro) | 2015 | 0.6 | 0.5 | 0.7 |
| Noncore (Nonmetro) | 2016 | 0.8 | 0.6 | 0.9 |
| Noncore (Nonmetro) | 2017 | 0.9 | 0.8 | 1.1 |
| Noncore (Nonmetro) | 2018 | 1.1 | 1 | 1.3 |
| Noncore (Nonmetro) | 2019 | 1.2 | 1 | 1.3 |
| Noncore (Nonmetro) | 2020 | 1.6 | 1.4 | 1.8 |
