# Supplementary material for: Demographic and Geographic Disparities in Atrial Fibrillation and Cirrhosis Mortality in the United States: A Twenty-Five-Year Analysis From 1999 to 2023
Source: Cardiol Res. 2026 Apr 15;17(2):105–19. doi: 10.14740/cr2194 (PMC13094160; doi:10.14740/cr2194)
Supplement: Suppl 15 — AAMR from 1999 to 2020. [file cr-17-02-105-s015.docx]

**Suppl 15.** AAMR from 1999 to 2020.

| State | Age Adjusted Rate | Age Adjusted Rate Lower 95% Confidence Interval | Age Adjusted Rate Upper 95% Confidence Interval |
| --- | --- | --- | --- |
| Alabama | 0.5 | 0.4 | 0.5 |
| Alaska | 0.7 | 0.5 | 0.9 |
| Arizona | 0.5 | 0.4 | 0.5 |
| Arkansas | 0.4 | 0.4 | 0.5 |
| California | 0.7 | 0.7 | 0.7 |
| Colorado | 0.7 | 0.6 | 0.7 |
| Connecticut | 0.5 | 0.4 | 0.5 |
| Delaware | 0.5 | 0.4 | 0.6 |
| District of Columbia | 0.4 | 0.3 | 0.6 |
| Florida | 0.4 | 0.4 | 0.4 |
| Georgia | 0.4 | 0.3 | 0.4 |
| Hawaii | 0.5 | 0.4 | 0.6 |
| Idaho | 0.7 | 0.6 | 0.8 |
| Illinois | 0.4 | 0.3 | 0.4 |
| Indiana | 0.6 | 0.5 | 0.6 |
| Iowa | 0.4 | 0.4 | 0.5 |
| Kansas | 0.3 | 0.3 | 0.4 |
| Kentucky | 0.7 | 0.6 | 0.8 |
| Louisiana | 0.3 | 0.3 | 0.4 |
| Maine | 0.6 | 0.5 | 0.7 |
| Maryland | 0.5 | 0.5 | 0.6 |
| Massachusetts | 0.5 | 0.4 | 0.5 |
| Michigan | 0.3 | 0.3 | 0.4 |
| Minnesota | 0.7 | 0.7 | 0.8 |
| Mississippi | 0.5 | 0.4 | 0.5 |
| Missouri | 0.4 | 0.3 | 0.4 |
| Montana | 0.4 | 0.3 | 0.5 |
| Nebraska | 0.6 | 0.5 | 0.7 |
| Nevada | 0.3 | 0.3 | 0.4 |
| New Hampshire | 0.6 | 0.5 | 0.8 |
| New Jersey | 0.5 | 0.4 | 0.5 |
| New Mexico | 0.5 | 0.4 | 0.6 |
| New York | 0.3 | 0.3 | 0.3 |
| North Carolina | 0.6 | 0.6 | 0.6 |
| North Dakota | 0.6 | 0.4 | 0.7 |
| Ohio | 0.6 | 0.6 | 0.7 |
| Oklahoma | 0.8 | 0.8 | 0.9 |
| Oregon | 0.9 | 0.8 | 0.9 |
| Pennsylvania | 0.6 | 0.5 | 0.6 |
| Rhode Island | 0.8 | 0.7 | 0.9 |
| South Carolina | 0.7 | 0.6 | 0.7 |
| South Dakota | 0.4 | 0.3 | 0.5 |
| Tennessee | 0.7 | 0.7 | 0.8 |
| Texas | 0.8 | 0.8 | 0.9 |
| Utah | 0.3 | 0.2 | 0.4 |
| Vermont | 0.9 | 0.7 | 1.1 |
| Virginia | 0.5 | 0.4 | 0.5 |
| Washington | 0.8 | 0.7 | 0.8 |
| West Virginia | 0.8 | 0.7 | 0.9 |
| Wisconsin | 0.5 | 0.4 | 0.5 |
| Wyoming | 0.6 | 0.4 | 0.7 |
