# Supplementary material for: Demographic and Geographic Disparities in Atrial Fibrillation and Cirrhosis Mortality in the United States: A Twenty-Five-Year Analysis From 1999 to 2023
Source: Cardiol Res. 2026 Apr 15;17(2):105–19. doi: 10.14740/cr2194 (PMC13094160; doi:10.14740/cr2194)
Supplement: Suppl 16 — Total deaths stratified by place of death from 1999 to 2023. [file cr-17-02-105-s016.docx]

**Suppl 16.** Total deaths stratified by place of death from 1999 to 2023.

| **Place of Death** | **Total Deaths** |
| --- | --- |
| Medical Facility – Inpatient | **17,996** |
| Medical Facility – Outpatient / ER | **1,267** |
| Medical Facility – Dead on Arrival | **55** |
| Decedent’s Home | **10,827** |
| Hospice Facility | **2,919** |
| Nursing Home / Long-Term Care | **5,605** |
